# Extracellular Vesicles from Child Gut Microbiota Enter into Bone to Preserve Bone Mass and Strength

**DOI:** 10.1002/advs.202004831

**Published:** 2021-02-17

**Authors:** Jiang‐Hua Liu, Chun‐Yuan Chen, Zheng‐Zhao Liu, Zhong‐Wei Luo, Shan‐Shan Rao, Ling Jin, Teng‐Fei Wan, Tao Yue, Yi‐Juan Tan, Hao Yin, Fei Yang, Fei‐Yu Huang, Jian Guo, Yi‐Yi Wang, Kun Xia, Jia Cao, Zhen‐Xing Wang, Chun‐Gu Hong, Ming‐Jie Luo, Xiong‐Ke Hu, Yi‐Wei Liu, Wei Du, Juan Luo, Yin Hu, Yan Zhang, Jie Huang, Hong‐Ming Li, Ben Wu, Hao‐Ming Liu, Tuan‐Hui Chen, Yu‐Xuan Qian, You‐You Li, Shi‐Kai Feng, Yang Chen, Lu‐Yue Qi, Ran Xu, Si‐Yuan Tang, Hui Xie

**Affiliations:** ^1^ Department of Orthopedics Xiangya Hospital Central South University Changsha Hunan 410008 China; ^2^ Movement System Injury and Repair Research Center Xiangya Hospital Central South University Changsha Hunan 410008 China; ^3^ Department of Sports Medicine Xiangya Hospital Central South University Changsha Hunan 410008 China; ^4^ National Clinical Research Center for Geriatric Disorders (Xiangya Hospital) Changsha Hunan 410008 China; ^5^ Xiangya Nursing School Central South University Changsha Hunan 4100113 China; ^6^ Department of Occupational and Environmental Health Xiangya School of Public Health Central South University Changsha Hunan 410078 China; ^7^ Department of Rehabilitation Xiangya Hospital Central South University Changsha Hunan 410008 China; ^8^ Department of Urology The Second Xiangya Hospital Central South University Changsha Hunan 410008 China; ^9^ Hunan Key Laboratory of Organ Injury Aging and Regenerative Medicine (Xiangya Hospital) Changsha Hunan 410008 China; ^10^ Hunan Key Laboratory of Bone Joint Degeneration and Injury (Xiangya Hospital) Changsha Hunan 410008 China

**Keywords:** *Akkermansia muciniphila*, bone homeostasis, extracellular vesicles, gut microbiota

## Abstract

Recently, the gut microbiota (GM) has been shown to be a regulator of bone homeostasis and the mechanisms by which GM modulates bone mass are still being investigated. Here, it is found that colonization with GM from children (CGM) but not from the elderly (EGM) prevents decreases in bone mass and bone strength in conventionally raised, ovariectomy (OVX)‐induced osteoporotic mice. 16S rRNA gene sequencing reveals that CGM reverses the OVX‐induced reduction of *Akkermansia muciniphila* (*Akk*). Direct replenishment of *Akk* is sufficient to correct the OVX‐induced imbalanced bone metabolism and protect against osteoporosis. Mechanistic studies show that the secretion of extracellular vesicles (EVs) is required for the CGM‐ and *Akk*‐induced bone protective effects and these nanovesicles can enter and accumulate into bone tissues to attenuate the OVX‐induced osteoporotic phenotypes by augmenting osteogenic activity and inhibiting osteoclast formation. The study identifies that gut bacterium *Akk* mediates the CGM‐induced anti‐osteoporotic effects and presents a novel mechanism underlying the exchange of signals between GM and host bone.

## Graphical abstract

Colonization with gut microbiota from children (CGM) but not from the elderly (EGM) reverses the ovariectomy (OVX)‐induced reduction of *Akkermansia muciniphila* (*Akk*) and prevents OVX‐induced osteoporosis. Direct replenishment of *Akk* also induces bone benefits in OVX mice. Extracellular vesicles from CGM and *Akk* can enter into bone to directly attenuate the OVX‐induced osteoporotic phenotypes by augmenting osteogenesis and inhibiting osteoclastogenesis.

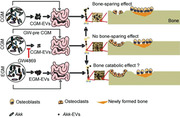

## 1 Introduction

Osteoporosis is a common bone disease in the elderly especially in postmenopausal women. More ideal interventions are still required to improve bone health and prevent osteoporosis. Gut microbiota (GM) is the community of microbes (bacteria, fungi, viruses, etc.) residing in the host gastrointestinal tract. The host gut provides an environment for GM to grow and members in GM such as some gut bacteria participate in maintaining host homeostasis by regulating various physiological processes such as gut development, nutrient digestion, immune status and brain function.^[^ ^1^ ^]^ Recent work has also linked GM to bone health. Sjögren et al. found that 7‐week‐old germ‐free female C57BL/6 mice have fewer osteoclasts and higher bone mass than age‐ and sex‐matched conventionally raised mice; bacterial colonization of germ‐free mice for 1 month normalizes bone mass,^[^ ^2^ ^]^ suggesting that GM has a negative effect on bone. However, a recent study by Quach et al. showed that colonization with mouse‐ or human‐derived GM does not cause bone loss in 4‐week‐old germ‐free male and female Swiss Webster mice and C57BL/6 mice.^[^ ^3^ ^]^ Schwarzer et al. showed that 8‐week‐old germ‐free male BALB/c mice exhibit slower bone growth relative to the conventionally raised mice.^[^ ^4^ ^]^ Yan et al. reported that long‐term colonization of 2‐month‐old germ‐free female and male CB6F1 mice with fecal microbiota from 3‐month‐old specific pathogen‐free male mice results in a significant increase in femur length and a trend of improvement of trabecular bone microarchitecture.^[^ ^5^ ^]^ Thus, GM also has a positive effect on bone. Discrepant results regarding the impact of GM on bone are possibly due to differences in mouse strain, age, sex, or duration of colonization.^[^ ^5^ ^]^ Another critical variable that may determine the effect of GM on bone is the composition of microbial community. There are evidences that bone mineral density (BMD) is increased when the conventional female C57BL/6 mice at weaning or at birth receive subtherapeutic antibiotic treatment for 3^[^ ^6^ ^]^ or 20^[^ ^7^ ^]^ weeks. Subtherapeutic antibiotic exposure does not reduce microbial abundance, but markedly alters microbial community composition and increases phylogenetic diversity in fecal microbiota of mice,^[^ ^7^ ^]^ suggesting that the changes of abundance of some gut microbes may contribute to the positive effect of subtherapeutic antibiotic treatment on bone mass. Previous studies have demonstrated the beneficial effects of many probiotic bacteria on bone health,^[^ ^4^, ^8^, ^9^ ^]^ suggesting that the influence of GM on bone is microbe specific. GM may consist of microbes beneficial or harmful for bone health and changes in GM community structure may result in a different impact of GM on bone.

GM composition can be affected by various factors such as estrogen deficiency and aging.^[^ ^10^ ^]^ Skeletal aging and estrogen deficiency can lead to an imbalance of bone remodeling characterized by bone resorption greater than bone formation, thereby causing bone loss and even osteoporosis.^[^ ^11^ ^]^ Considering that GM in different studies has been shown to exert different effects on bone health and the discrepancy may be associated with the shifts of GM composition, we hypothesized that the changes of some microbe abundance may be a critical factor that induces an imbalance in bone remodeling and subsequent bone loss. Thus, what becomes particularly important is the identification of microbes regulating this process and how to restore a balanced GM to prevent osteoporosis. It has been reported that transplantation with fecal microbiota from lean humans reduces adiposity gain in the obese‐recipient mice;^[^ ^12^ ^]^ the mice with a Malawian diet become undernourished after receiving fecal microbiota from the undernourished Malawian infants/children,^[^ ^13^ ^]^ suggesting the potential of GM to transfer some phenotypes from the donors to the recipients. As childhood is a time of rapid growth, we speculated that GM from healthy children may contain specific microbes beneficial to bone health, and transplantation with either children GM or the beneficial microbes may be able to restore bone remodeling balance and to induce a healthy bone phenotype in the osteoporotic recipients.

Given the importance of GM in modulating bone health status, it becomes meaningful to explore the mechanism through which GM communicates with the host bone. In recent years, it has been recognized that the exchange of biological signals between cells in both prokaryotes and higher eukaryotes is largely mediated by the secretion of extracellular vesicles (EVs).^[^ ^14^ ^]^ EVs production is ubiquitously present in Gram‐negative bacteria and in some Gram‐positive bacteria.^[^ ^15^, ^16^, ^17^ ^]^ GM‐derived EVs (20–400 nm) enable bacteria to deliver a multitude of effector molecules to distant target cells in a concentrated and protected manner, thereby regulating the function of recipient cells.^[^ ^15^, ^16^, ^17^ ^]^ In 2013, Kang et al. characterized stool‐derived EVs and found that EVs from a member of stool bacteria, *Akkermansia muciniphila* (*Akk*), can inhibit inflammation in colon epithelial cells induced by *Escherichia coli*‐derived EVs and protect mice from experimental colitis,^[^ ^18^ ^]^ suggesting a functional role of EVs from a specific member of GM. Recently, Choi et al. reported that the oral application of stool‐derived EVs from high fat diet‐fed mice or EVs from the gut commensal *Pseudomonas panacis* can induce insulin resistance and glucose intolerance in recipient mice.^[^ ^19^ ^]^ Moreover, they found that *P. panacis*‐EVs may cross the mouse intestinal epithelial barrier and enter into the insulin‐responsive tissues such as liver, adipose tissue, and skeletal muscle,^[^ ^19^ ^]^ implying that GM can regulate the function of distal organs in the host by EV‐based direct interaction with the targets. This prompts us to explore whether the transfer of functional EVs from specific microbes to bone tissues is a key mechanism of the GM‐induced regulation of bone health.

Here, we explored whether colonization of conventionally raised, ovariectomy (OVX)‐induced osteoporotic C57BL/6 mice with GM from healthy children can induce bone growth and protect against osteoporosis by reversing the levels of specific gut microbes beneficial to bone health. We also determined whether the distal transfer of functional EVs from these specific microbes to bone cells is a key mechanism by which GM regulates bone mass.

## 2 Results

### 2.1 GM from Healthy Children (CGM) Prevents Bone Loss and Alters Bone Metabolism in OVX Mice

To explore whether GM can prevent bone loss of osteoporotic recipients, we established an animal model of postmenopausal osteoporosis by OVX surgery in conventionally raised C57BL/6 mice. We harvested CGM and EGM samples, respectively, from three healthy children and three healthy old people from three different families (one CGM donor and one EGM donor per family). Table S1 in the Supporting Information shows the levels of parameters revealing the metabolic status of the donor subjects, including the values of body weight, height, body mass index (BMI; weight/height^2^, kg m^−2^), blood glucose, serum total protein, albumin, globulin, albumin/globulin ratio, total cholesterol, and triglyceride. These parameters in CGM and EGM donors were mainly fluctuated within the range of normal level (Table S1, Supporting Information), suggesting that all CGM and EGM donors are in a healthy metabolic status. The EGM donors had higher average values of blood glucose, serum total protein, globulin, total cholesterol, and triglyceride, and lower values of serum albumin and albumin/globulin ratio compared with the CGM donors, but only by trend (Table S1, Supporting Information).

**Figure 1:**
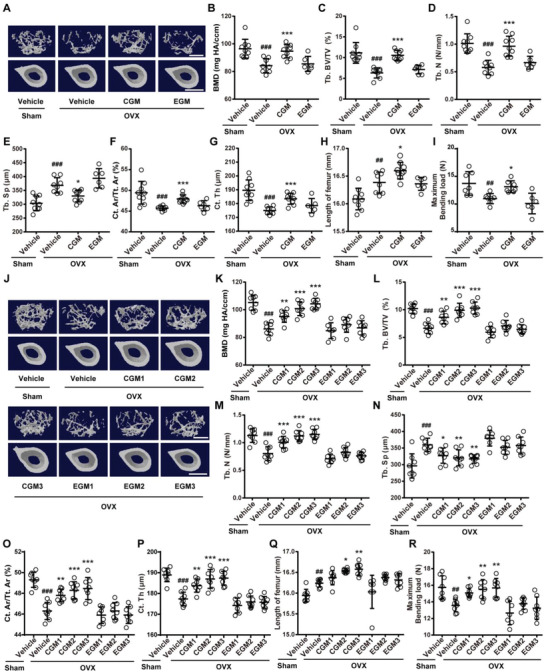
CGM increases bone mass, bone length, and bone strength in OVX mice. A) Representative µCT images of trabecular (up) and cortical (bottom) bone in femora from mice in Sham + Vehicle, OVX + Vehicle, OVX + pooled CGM, and OVX + pooled EGM groups. CGM: gut microbiota from child. EGM: gut microbiota from the elderly. Scale bars: 500 µm (up) and 1 mm (bottom). Quantitative analysis of B) bone mineral density (BMD), C) trabecular bone volume fraction (Tb. BV/TV), D) trabecular number (Tb. N), E) trabecular separation (Tb. Sp), F) cortical bone area fraction (Ct. Ar/Tt. Ar), and G) cortical thickness (Ct. Th). *n* = 6–9 per group. H) Lengths of femora. *n* = 6–9 per group. I) Three‐point bending measurement of femur ultimate load. *n* = 6–7 per group. J) Representative µCT images of trabecular (up) and cortical (bottom) bone in femora from Sham and OVX mice treated with vehicle or different donor‐derived CGM or EGM. Scale bars: 500 µm (up) and 1 mm (bottom). Quantitative analysis of K) BMD, L) Tb. BV/TV, M) Tb. N, N) Tb. Sp, O) Ct. Ar/Tt. Ar, and P) Ct. Th. *n* = 7–8 per group. Q) Lengths of femora. *n* = 7–8 per group. R) Femur ultimate load measured by three‐point bending test. *n* = 7–8 per group. Data are presented as mean ± SD. ^**#**^ *P* < 0.05 versus Sham + Vehicle group and * *P* < 0.05 versus OVX + Vehicle group. * *P* < 0.05, ^##/**^ *P* < 0.01, and ^###/***^ *P* < 0.001.

We mixed the different donor‐derived CGM or EGM equally and investigated the effects of the mixture of CGM or EGM on bone metabolism. Figure S1A in the Supporting Information shows that all OVX mice had increased body weights compared to Sham‐operated (Sham) mice, consistent with the results of previous studies.^[^ ^20^ ^]^ The success of OVX was confirmed by decreased uterus weights and uterus sizes in all OVX mice compared with Sham mice (Figure S1B,C, Supporting Information). Microcomputed tomography (µCT) analysis of femurs showed that the vehicle‐treated OVX mice exhibited markedly decreased bone mass and impaired bone microstructures relative to Sham mice, as indicated by significantly lower BMD, trabecular bone volume fraction (Tb. BV/TV), trabecular number (Tb. N), trabecular thickness (Tb. Th), cortical bone area fraction (Ct. Ar/Tt. Ar), and cortical thickness (Ct. Th), a trend of decrease of cortical bone area (Ct. Ar), and significantly higher trabecular separation (Tb. Sp) than that in Sham mice (**Figure** **1**A–G; Figure S1D–F, Supporting Information). After an 8‐week twice weekly oral administration of the pooled CGM, but not EGM, most of these altered parameters revealing bone mass or bone microstructures were significantly reversed (Figure 1A–G; Figure S1D–F, Supporting Information). Consistent with the rescued bone mass and bone microstructures, femur length analysis and three‐point bending test indicated that colonization with the pooled CGM resulted in a further increase in femur length and blocked the reduction of maximum bending load of the femurs in OVX mice, whereas EGM treatment caused trends of decreases in bone length and strength (Figure 1H,I). To exclude the possibility of individual differences, we then obtained fresh CGM and EGM samples from the above‐described donors and did not mix either CGM or EGM samples from different donors. Significant increase of body weights (Figure S2A, Supporting Information) and decrease of uterus weights (Figure S2B, Supporting Information) were observed in all OVX mice compared with Sham mice. We performed animal experiments to assess the impacts of each of CGM and EGM from these donors on bone health. The results showed that all child donor‐derived GM, similar to the pooled GM from different child donors, could independently inhibit the loss of bone mass in OVX mice, but none of EGM from the above‐described donors induced notable bone protective effects (Figure 1J–P; Figure S2C–E, Supporting Information). Moreover, the OVX mice treated with the donor 2‐ and donor‐3‐derived CGM (CGM2 and CGM3) exhibited markedly increased femur lengths compared to the vehicle‐treated OVX mice, but the effect was not detected in OVX mice treated with each of EGM from different donors (Figure 1Q). All CGM, but not any of these EGM, also significantly reversed the OVX‐induced reduction of maximum bending load of the femurs (Figure 1R). These results suggest that transplanting GM from young donors can inhibit bone loss, enhance bone growth, and maintain bone strength in OVX mice, and the anti‐osteoporotic effects of GM are lost with donor aging.

**Figure 2:**
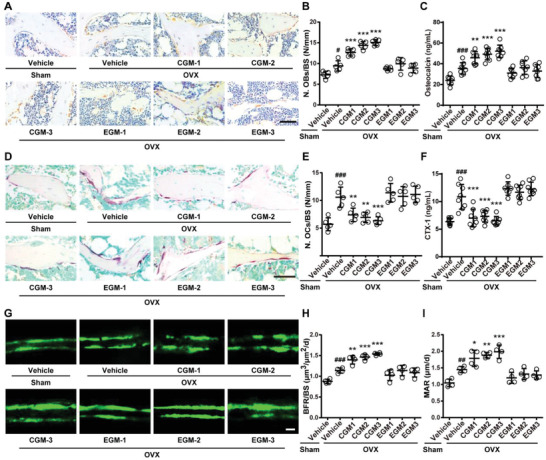
CGM reverses the imbalanced bone metabolism in OVX mice. A) Representative OCN‐stained sections with quantification of the B) number of osteoblasts (N. OBs) on trabecular bone surface (BS) in distal femora from mice treated with vehicle or different donor‐derived CGM or EGM. *n* = 5 per group. Scale bar: 50 µm. C) ELISA for serum OCN. *n* = 7–8 per group. D) Representative TRAP‐stained sections with quantitation of the E) number of osteoclasts (N. OCs). *n* = 5 per group. Scale bar: 50 µm. F) ELISA for serum CTX‐I. *n* = 7–8 per group. G) Representative images of calcein double labeling with quantitation of H) bone formation rate per bone surface (BFR/BS) and I) mineral apposition rate (MAR). Scale bar: 10 µm. *n* = 4 per group. Data are presented as mean ± SD. **^#^** *P* < 0.05 versus Sham + Vehicle group and * *P* < 0.05 versus OVX + Vehicle group. ^#/*^ *P* < 0.05, ^##/**^ *P* < 0.01, and ^###/***^ *P* < 0.001.

We then tested the impacts of different donor‐derived CGM and EGM on osteogenesis and osteoclastogenesis in OVX mice. OCN immunohistochemical staining showed greater number of osteoblasts on trabecular bone surface in the vehicle‐treated OVX mice compared with Sham mice, and colonization of OVX mice with each of the above‐described child donor‐derived GM further increased the number of osteoblasts (**Figure** **2**A,B). However, the pro‐osteogenic effect was not observed in OVX mice treated with either of the obtained EGM (Figure 2A,B). Consistently, enzyme‐linked immunosorbent assay (ELISA) revealed that colonization with different donor‐derived CGM, but not EGM, further augmented the serum level of osteocalcin (OCN, a marker of osteogenic differentiation^[^ ^21^, ^22^ ^]^) in OVX mice (Figure 2C). Tartrate‐resistant acid phosphatase (TRAP) staining indicated that OVX induced marked increases in the number and size of osteoclasts, but the effects were entirely reversed by colonization with different donor‐derived CGM (Figure 2D,E; Figure S2F, Supporting Information). No notable changes in osteoclast number and size were observed in all EGM‐colonized OVX mice (Figure 2D,E; Figure S2F, Supporting Information). ELISA showed that all donor‐derived CGM significantly suppressed the OVX‐induced increase of serum level of C‐terminal telopeptides of type I collagen (CTX‐I), whereas the EGM‐treated OVX mice exhibited a trend of further increase in the serum level of this bone resorption marker compared with the vehicle‐treated OVX mice (Figure 2F), suggesting a catabolic impact of EGM on bone. Calcein double labeling revealed that transplantation with each of different donor‐derived CGM could rescue the impairment of new bone formation and mineralization in OVX mice, while all donor‐derived EGM failed to affect this process, as indicated by bone formation rate per bone surface (BFR/BS) and mineral apposition rate (MAR) values (Figure 2G–I). These findings suggest that colonization with CGM to osteoporotic mice reverses the imbalanced bone metabolism by promoting osteoblastic bone formation and suppressing osteoclastic bone resorption, which finally enhances bone mass and strength.

### 2.2 CGM Rescues the Loss of Gut *A. muciniphila* (*Akk*) during Osteoporosis

**Figure 3:**
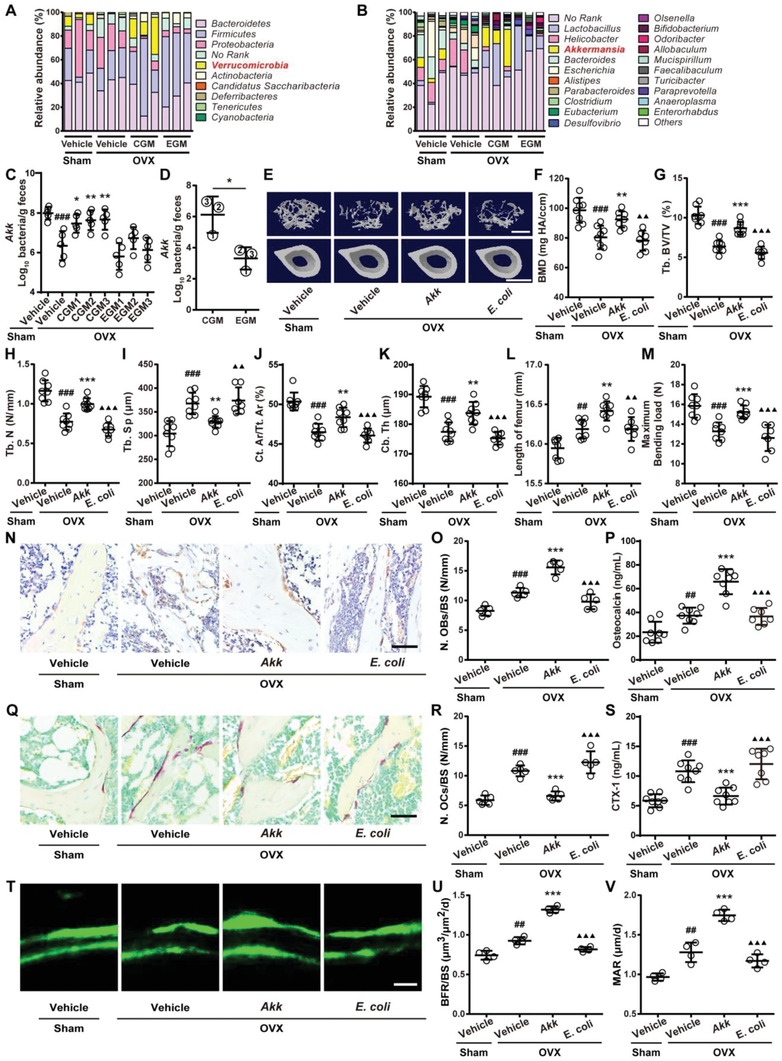
CGM rescues the loss of gut *Akk* during osteoporosis and supplementation of *Akk* attenuates osteoporotic phenotypes in OVX mice. Composition of fecal microbiota at the A) phylum and B) genus levels tested by 16S rRNA gene sequencing. *n* = 3 per group. As for genus, the proportion <1% occupancy is noted as others. qRT‐PCR analysis of *Akk* abundance in fecal microbiota from C) Sham and OVX mice receiving different treatments (*n* = 5 per group), or from D) different CGM and EGM donors (*n* = 3 per group). E) Representative µCT images of trabecular (up) and cortical (bottom) bone in femora and quantification of F) BMD, G) Tb. BV/TV, H) Tb. N, I) Tb. Sp, J) Ct. Ar/Tt. Ar, and K) Ct. Th. Scale bars: 500 µm (up) and 1 mm (bottom). *n* = 7–8 per group. L) Lengths of femora. *n* = 7–8 per group. M) Femur ultimate load measured by three‐point bending test. *n* = 7–8 per group. N) Representative OCN‐stained sections with quantification of O) osteoblast number. Scale bar: 50 µm. *n* = 5 per group. P) ELISA for serum OCN. *n* = 7–8 per group. Q) Representative TRAP‐stained sections with quantification of R) osteoclast number. Scale bar: 50 µm. *n* = 5 per group. S) ELISA for serum CTX‐I. *n* = 7–8 per group. T) Representative images of calcein double labeling with quantitation of U) BFR/BS and V) MAR. Scale bar: 10 µm. *n* = 4 per group. Data are presented as mean ± SD. For panel (D): * *P* < 0.05 versus CGM group. For other dot plots: **^#^** *P* < 0.05 versus Sham + Vehicle group, * *P* < 0.05 versus OVX + Vehicle group, and **^▲^** *P* < 0.05 versus OVX + *Akk* group. * *P* < 0.05, ^##/**/▲▲^ *P* < 0.01, and ^###/***/▲▲▲^ *P* < 0.001.

To find out the beneficial microbe that mediates the bone‐beneficial effects of CGM, we collected stool specimens from mice in Sham + Vehicle, OVX + Vehicle, OVX + pooled CGM, and OVX + pooled EGM groups, and evaluated the microbial composition in these specimens by high‐throughput sequencing of 16S rRNA gene (16S rDNA) amplicons. CGM and EGM samples from three different CGM and EGM donors were also obtained for the relative quantification of microbiota abundance using 16S rRNA gene sequencing. The composition of the identified microbiota was profiled at the levels of phylum, class, order, family, genus, and species (**Figure** **3**A,B; Tables S2 and S3, Supporting Information). At the phylum level, OVX induced significant decreases in *Verrucomicrobia* and *Deferribacteres*, and marked increases in *Candidatus Saccharibacteria* and *Tenericutes* relative to the Sham controls (Figure 3A; Table S2, Supporting Information). Colonization of OVX mice no matter with CGM or with EGM resulted in increases in *Actinobacteria* and *Firmicutes* (Figure 3A; Table S2, Supporting Information), which may be due to the difference in the microbial community structure of GM between mouse and human. The hypothesis was confirmed by the data of 16S rRNA gene sequencing for CGM and EGM samples, which showed higher abundance of bacteria in the phylum *Actinobacteria* and *Firmicutes* in CGM and EGM samples compared to the mouse fecal microbiota (Table S3, Supporting Information). Surprisingly and most importantly, the OVX‐induced loss of phylum *Verrucomicrobia* was entirely rescued by colonization with CGM, but not EGM (Figure 3A; Table S2, Supporting Information), implying that restoring the levels of bacteria in the phylum *Verrucomicrobia* may contribute to the CGM‐induced bone benefits. At the genus level, *Akkermansia*, a newly identified genus in the phylum *Verrucomicrobia*,^[^ ^23^ ^]^ was found to be inhibited by OVX and reversed by CGM treatment, whereas other genera in this phylum were not detected in the fecal samples from above‐described mice (Figure 3B; Table S2, Supporting Information). At the species level, only *Akk* was detected in the genus *Akkermansia*, and exhibited a dramatic reduction after OVX and a significant restoration in OVX mice with CGM transplantation (Figure 3B; Table S2, Supporting Information). Quantitative real‐time PCR (qRT‐PCR) analysis targeting the variable regions of the 16S rRNA gene of *Akk* demonstrated that each of the different donor‐derived CGM, similar to the pooled CGM from these donors, could profoundly reverse the reduction of gut bacterium *Akk* induced by OVX, whereas each of EGM from different donors did not restore *Akk* abundance in OVX mice (Figure 3C). 16S rRNA gene sequencing for CGM and EGM samples revealed that CGM donors had higher levels of *Akk* than EGM donors (Table S3, Supporting Information). Consistently, qRT‐PCR confirmed that the levels of *Akk* in fecal microbiota from EGM donors were much lower than that from CGM donors (Figure 3D). CGM1, which exhibited a trend of lower ability to induce bone benefits compared with CGM2 and CGM3, showed the lowest level of *Akk* among the different donor‐derived CGM (Figure 3D). These findings suggest that the loss of *Akk* is probably correlated with OVX‐induced osteoporosis and the replenishment of this microbe may be a critical mechanism by which CGM prevents osteoporosis.

### 2.3 Supplementation of *Akk* Attenuates Osteoporotic Phenotypes in OVX Mice

We next determined whether the direct supplementation of *Akk* twice a week for 8 weeks is sufficient to prevent OVX‐induced osteoporosis in mice and compared the effects of *Akk* on bone with *E. coli*, a bacterium that commonly resides in the gut of humans and warm‐blooded animals^[^ ^24^ ^]^ and was also present in fecal microbiota of all the vehicle‐treated Sham mice in the present study (Table S2, Supporting Information). µCT analysis revealed that the oral administration of the live *Akk* could block the OVX‐induced loss of bone mass, and improved bone microarchitecture, as indicated by the increased BMD, Tb. BV/TV, Tb. N, Ct. Ar/Tt. Ar, and Ct. Th, as well as the decreased Tb. Sp compared to the vehicle‐treated OVX mice (Figure 3E–K). Treatment with the live *Akk* also significantly reversed the OVX‐induced reduction of Ct. Ar, without notable effects on Tb. Th and total cross‐sectional area (Tt. Ar) (Figure S3A–C, Supporting Information). However, *E. coli* induced no obvious effects on bone mass and bone microstructures in OVX mice (Figure 3E–K; Figure S3A–C, Supporting Information). Consistently, bone length analysis and three‐point bending test, respectively, indicated that *Akk*, but not *E. coli*, was capable of increasing bone length and restoring bone strength in OVX mice (Figure 3L,M).

Next, we tested whether *Akk* can increase osteogenesis and decrease osteoclastogenesis like CGM. OCN immunohistochemical staining and ELISA test for this bone formation marker indicated that transplantation with *Akk* resulted in great increases in osteogenic responses in OVX mice compared to the vehicle‐treated OVX mice (Figure 3N–P). *E. coli*, however, induced a trend of decrease in osteoblast number and did not affect the serum level of OCN in OVX mice (Figure 3N–P). TRAP staining and ELISA for CTX‐I revealed that the administration of *Akk*, but not *E. coli*, inhibited the OVX‐induced promotion of osteoclast formation, osteoclast size and bone resorption activity (Figure 3Q–S; Figure S3D, Supporting Information). Calcein double labeling showed increased new bone formation and mineralization in OVX mice after treatment with *Akk*, whereas *E. coli* caused trend of decreases in BFR/BS and MAR values compared to the vehicle‐treated OVX mice (Figure 3T–V). The above results suggest that the gut bacterium *Akk*‐mediated bone‐beneficial effects by augmenting osteogenesis and reducing osteoclastogenesis may contribute importantly to the benefits of CGM on bone.

### 2.4 EVs Secretion Is Required for the Anti‐osteoporotic Activity of CGM and *Akk*

**Figure 4:**
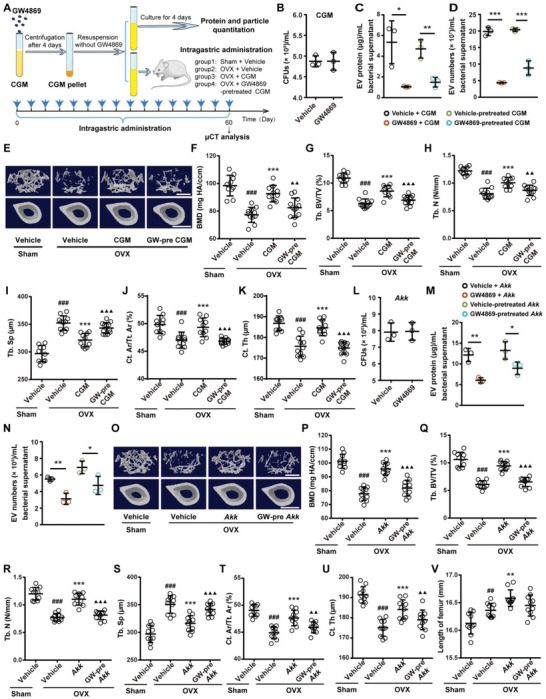
EVs secretion is required for the anti‐osteoporotic activity of CGM and *Akk*. A) Experiment design for testing the impact of GW4869 on the secretion of EVs by CGM and on the anti‐osteoporotic properties of CGM. The Sham and OVX mice were orally treated with vehicle, CGM, or GW4869‐pretreated CGM twice a week for 8 weeks (16 times within 60 days). B) Quantification of the number of bacterial colonies formed by the vehicle‐ or GW4869‐treated CGM. *n* = 3 per group. C) Total protein contents and D) particle numbers of EVs from CGM treated with vehicle or GW4869 for 4 days, or from CGM pretreated with vehicle or GW4869 for 4 days and cultured in fresh medium without vehicle or GW4869 for another 4 days. *n* = 3 per group. E) Representative µCT images of trabecular (up) and cortical (bottom) bone in femora and quantification of F) BMD, G) Tb. BV/TV, H) Tb. N, I) Tb. Sp, J) Ct. Ar/Tt. Ar, and K) Ct. Th. Scale bars: 500 µm (up) and 1 mm (bottom). *n* = 10–12 per group. L) Quantification of the number of bacterial colonies formed by the vehicle‐ or GW4869‐treated *Akk*. *n* = 3 per group. M) Total protein contents and N) particle numbers of EVs from *Akk* treated or pretreated with vehicle or GW4869 for 4 days. *n* = 3 per group. O) Representative µCT images of trabecular (up) and cortical (bottom) bone in femora and quantification of P) BMD, Q) Tb. BV/TV, R) Tb. N, S) Tb. Sp, T) Ct. Ar/Tt. Ar, and U) Ct. Th. Scale bars: 500 µm (up) and 1 mm (bottom). *n* = 10 per group. V) Lengths of femora. *n* = 10 per group. Data are presented as mean ± SD. For panels (F)–(J) and (O)–(T): **^#^** *P* < 0.05 versus Sham + Vehicle group, * *P* < 0.05 versus OVX + Vehicle group, and **^▲^** *P* < 0.05 versus OVX + CGM or *Akk* group. * *P* < 0.05, ^##/**/▲▲^ *P* < 0.01, and ^###/***/▲▲▲^ *P* < 0.001.

To investigate the involvement of EVs in the CGM‐induced anti‐osteoporotic effects, we preincubated CGM from donor 1 with GW4869, a neutral sphingomyelinase (nSMase) inhibitor that is able to impair the release of EVs,^[^ ^25^ ^]^ to interfere with the secretion of EVs by CGM. Then, we assessed whether the intragastric administration of these GW4869‐pretreated CGM twice a week for 8 weeks can protect against OVX‐induced osteoporosis in mice. The schematic diagram of the experimental procedures was shown in **Figure** **4**A. Bacterial colony counting assay on YCFA agar plates was performed to assess the effect of GW4869 on viability of CGM. As shown in Figure 4B, there was no statistically significant difference in the number of bacterial colonies between the GW4869‐ and vehicle‐treated CGM after plating on YCFA agar plates for 4 days, suggesting that the viability of bacteria is not impaired by GW4869. The EV protein represents an indicator of the number of EVs and the normalization of EV level to the concentration of total EV proteins is a simple and widely used method for quantifying EVs.^[^ ^15^, ^18^, ^19^, ^26^ ^]^ As shown in Figure 4C, the production of EVs was significantly inhibited in CGM after treatment with GW4869 for 4 days, as indicated by the significant reduction in total protein contents of isolated EVs. Even though GW4869 was removed from the medium and the treated CGM was cultured for another 4 days, the protein contents of EVs in the GW4869‐pretreated CGM were still much lower than that in the vehicle‐pretreated group (Figure 4C). We also used nanoparticle tracking analysis (NTA) to determine the particle number of EVs from CGM treated with GW4869 or vehicle. Consistent with the changes of the protein contents of EVs, the particle number of EVs was markedly decreased in CGM after treatment with GW4869 for 4 days and the reductions were still observed when GW4869 was removed from the cultures for 4 days (Figure 4D). These results indicate a lasting inhibitory effect of GW4869 on EV secretion. Strikingly, µCT analysis revealed that the abilities of CGM to improve bone mass and bone microarchitecture in OVX mice, including the up‐regulation of BMD, Tb. BV/TV, Tb. N, Tb. Th, Ct. Ar/Tt. Ar, Ct. Th, and Ct. Ar, as well as the downregulation of Tb. Sp, were almost entirely abolished by GW4869 pretreatment, as indicated by comparable levels of these bone mass and microstructural parameters between GW4869‐pretreated CGM‐ and vehicle‐treated OVX mice (Figure 4E–K; Figure S4A–C, Supporting Information), suggesting that the secretion of EVs is required for the bone protective effects of CGM.

We then determined whether EV production is essential for the bone‐beneficial effects of *Akk* by using GW4869. Treatment with GW4869 did not affect the viability of *Akk*, as revealed by bacterial colony counting assay on brain‐heart‐infusion (BHI) agar plate containing 0.05% l‐cysteine‐HCl and 0.5% porcine mucin (Figure 4L). The inhibition of GW4869 on EV production by *Akk* was confirmed by the significant reduction of total protein contents and particle numbers of EVs from *Akk* (*Akk*‐EVs) when their parent bacterium *Akk* was treated or pretreated with GW4869 (Figure 4M,N). Consistent with that observed in OVX mice treated with the GW4869‐preincubated CGM, µCT analysis showed that the pretreatment of *Akk* with GW4869 also abolished the ability of *Akk* to prevent the OVX‐induced loss of bone mass and impairment of bone microstructures (Figure 4O–U; Figure S4D–F, Supporting Information). Femur length analysis revealed that the stimulatory effect of *Akk* on longitudinal growth of bone was also blocked by GW4869 pretreatment (Figure 4V). These results suggest that EVs are the major effector of the *Akk*‐induced anti‐osteoporotic activity.

Since previous study by Lawenius et al. showed that treatment with pasteurized *Akk* cannot protect mice from OVX‐induced bone loss,^[^ ^27^ ^]^ we explored whether the process of pasteurization (70 °C, 30 min), a strategy frequently used for reducing microbial population in liquids and dairy items,^[^ ^28^ ^]^ impairs the production of EVs by *Akk* due to the death of *Akk*. Bacterial colony counting assay on agar plate was performed to assess *Akk* viability and the particle numbers of *Akk*‐EVs from pasteurized or live *Akk* were measured by nanoparticle tracking analysis. As shown in Figure S5A,B in the Supporting Information, pasteurization caused 100% death of *Akk* and entirely blocked the secretion of EVs by *Akk*, suggesting that the failure of pasteurized *Akk* to induce bone‐protective effects in study by Lawenius et al.^[^ ^27^ ^]^ may be associated with the impairment of production of *Akk*‐EVs.

### 2.5 EVs from CGM, EGM, or *Akk* Are Transported to Bone Tissues

**Figure 5:**
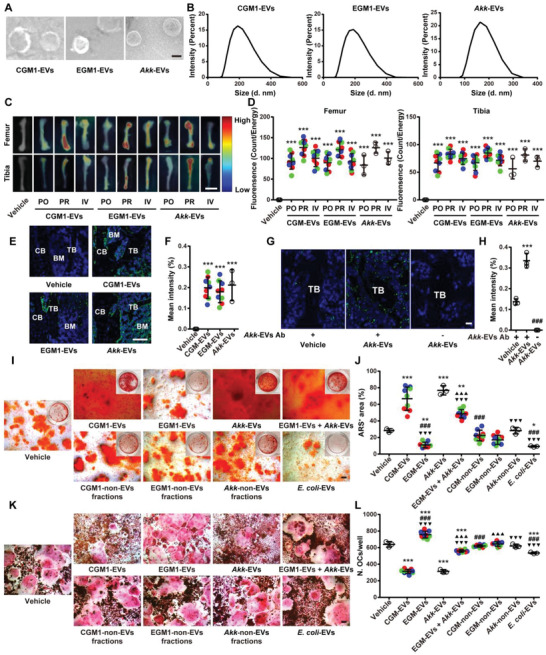
CGM‐EVs and *Akk*‐EVs can be transported to the mouse bone tissues and directly promote osteogenesis and inhibit osteoclastogenesis in vitro. A) Morphological analysis of CGM1‐EVs, EGM1‐EVs, and *Akk*‐EVs by transmission electron microscopy. Scale bar: 50 nm. B) Particle size distribution of different EVs measured by DLS. C) *Ex vivo* fluorescent imaging of the femur and tibia from mice treated with vehicle or the DIR‐labeled EVs for 1 h and D) quantification of the fluorescent signals. PO: *per os*; PR: *per rectal*; IV: intravenous. Scale bar: 6 mm. *n* = 3 per group. E) Confocal microscopy analysis of the femoral sections from mice treated with the PKH67‐labeled EVs for 1 h by oral route. CB: cortical bone; TB: trabecular bone; BM: bone marrow. Scale bar: 20 µm. F) Quantification of the fluorescent signals in (E). *n* = 3 per group. G) Representative images of the *Akk*‐EVs antibody (Ab)‐stained femoral sections with quantification of the H) positive signals. Scale bar: 10 µm. *n* = 3 per group. I) Representative ARS staining images of BMSCs receiving different treatments and J) quantitation of the percentage of ARS‐positive areas per well in a 48‐well plate. Scale bar: 100 µm. *n* = 3 per group. K) Representative TRAP staining images of RAW264.7 cells receiving different treatments and L) quantification of osteoclast number per well in a 48‐well plate. Scale bar: 100 µm. *n* = 3 per group. Data are presented as mean ± SD. For panels (D), (F), (J), and (L): Red dots indicate CGM1‐EVs or EGM1‐EVs, green dots indicate CGM2‐EVs or EGM2‐EVs, and blue dots indicate CGM3‐EVs or EGM3‐EVs. For panels (D) and (F): * *P* < 0.05 versus Vehicle group. For panel (H): * *P* < 0.05 versus Vehicle + *Akk*‐EVs Ab group and ^**#**^ *P* < 0.05 versus *Akk*‐EVs + *Akk*‐EVs Ab group. For panels (J) and (L): * *P* < 0.05 versus Vehicle group, **^#^** *P* < 0.05 versus CGM‐EVs group, **^▲^** *P* < 0.05 versus EGM‐EVs group, and **^▼^** *P* < 0.05 versus *Akk*‐EVs group. ^**^ *P* < 0.01 and ^***/###/▲▲▲/▼▼▼^ *P* < 0.001.

Next, we isolated EVs from the culture media of CGM (CGM1, CGM2, or CGM3), EGM (EGM1, EGM2, or EGM3), or *Akk* and characterized the isolated EVs using transmission electron microscopy and dynamic light scattering (DLS) analysis. As shown in **Figure** **5**A,B, CGM1‐derived EVs (CGM1‐EVs), EGM1‐derived EVs (EGM1‐EVs), and *Akk*‐EVs displayed cup‐shaped or spherical morphologies with diameters of 207.25 ± 54.23 nm, 193.99 ± 57.66 nm, and 172.81 ± 27.80 nm, respectively, similar to that previously reported EVs from bacteria.^[^ ^15^, ^16^, ^29^ ^]^ Then, we labeled three different donor‐derived CGM‐EVs (CGM1‐EVs, CGM2‐EVs, and CGM3‐EVs), three different donor‐derived EGM‐EVs (EGM1‐EVs, EGM2‐EVs, and EGM3‐EVs), and *Akk*‐EVs with DIR iodide and then examined whether these EVs can be transported to the host tissues. As revealed by *ex vivo* fluorescent imaging, high fluorescent signals were detected in the tibia and femur of mice orally, rectally or intravenously administered with the DIR‐labeled CGM‐EVs, EGM‐EVs, or *Akk*‐EVs for 1 h (Figure 5C,D), indicating that a large number of these bacterial EVs entered into the host bone tissues. The fluorescent signals were also detected in liver, kidney, brain, muscle, and gastrointestine, among which liver, muscle and gastrointestine showed the highest fluorescence intensity (Figure S6A,B, Supporting Information). These EVs were also labeled with a green fluorescent dye (PKH67). A confocal microscope was employed to detect the fluorescent signals in different tissues from mice orally treated with the labeled EVs for 1 h. Figure S6C,D in the Supporting Information shows that all CGM‐EVs, all EGM‐EVs, and *Akk*‐EVs were successfully transported to the mouse liver, kidney and brain. Figure 5E shows the presence of the PKH67‐labeled CGM1‐EVs, EGM1‐EVs, and *Akk*‐EVs in the mouse bone tissues, including the sites of periosteum, endosteum, cortical bone, trabecular bone, and bone marrow in mice, suggesting that these EVs may have the ability to directly target bone cells to modulate bone metabolism. Quantification of the mean fluorescent intensity confirmed that all CGM‐EVs, all EGM‐EVs, and *Akk*‐EVs exhibit comparable capacities to be transported to the mouse bone tissues (Figure 5F).

We also obtained serum of the *Akk*‐EV‐immunized rabbit and assessed whether the orally administered *Akk*‐EVs could be detected in the mouse tissues using the *Akk*‐EVs‐antibody‐containing serum. As shown in Figure 5G,H and Figure S6E,F in the Supporting Information, both the vehicle‐ and *Akk*‐EV‐treated mice showed fluorescent signals in bone, brain, liver, and kidney, and the signals in these tissues were much higher in the *Akk*‐EV‐treated mice compared with the vehicle‐treated control mice, suggesting that *Akk*‐EVs can be transported to the host tissues under physiological conditions and the oral treatment of exogenous *Akk*‐EVs further increases their abundance in these tissues.

### 2.6 CGM‐EVs and *Akk*‐EVs Promote Osteogenesis and Inhibit Osteoclastogenesis *In Vitro*

We next asked whether three different donor‐derived CGM‐EVs (CGM1‐EVs, CGM2‐EVs, and CGM3‐EVs), three different donor‐derived EGM‐EVs (EGM1‐EVs, EGM2‐EVs, and EGM3‐EVs), and *Akk*‐EVs are able to directly interact with bone cells*in vitro* and compared the abilities of CGM‐EVs and *Akk*‐EVs to modulate osteogenesis and osteoclastogenesis with EGM‐EVs, EGM‐EVs + *Akk*‐EVs, non‐EV‐containing fractions (obtained after density gradient centrifugation of CGM‐EVs, EGM‐EVs and *Akk*‐EVs), and *E. coli*‐derived EVs (*E. coli*‐EVs). As previous studies have shown that 10 µg mL^−1^ bacterial EVs are sufficient to induce significant effects in recipient cells *in vitro* ^[^ ^30^ ^]^ and we found that the particle numbers in 100 µg CGM‐EVs and *Akk*‐EVs from different batches were mostly at the range of (3–7) × 10^9^ vesicles ((5.27 ± 1.91) × 10^9^ vesicles) and (5–7) × 10^9^ vesicles ((6.00 ± 1.13) × 10^9^ vesicles), respectively (Figure S7A, Supporting Information), the dose of 6 × 10^8^ vesicles mL^−1^ (≈10 µg mL^−1^ protein contents) was chosen for all EVs‐related assays *in vitro*. The non‐EV‐containing fractions were used at the concentration of 10 µg mL^−1^ protein contents.

The fluorescence microscope showed that the PKH26‐labeled three CGM‐EVs, three EGM‐EVs, and *Akk*‐EVs could enter into the perinuclear region of mouse primary bone marrow mesenchymal stem cells (BMSCs) and osteoclast progenitor RAW264.7 cells after 3 h incubation (Figure S7B,C, Supporting Information). Alizarin Red S (ARS) staining revealed that BMSCs treated with CGM1‐EVs, CGM2‐EVs, CGM3‐EVs, or *Akk*‐EVs showed much higher levels of calcium nodule formation compared with the vehicle‐treated BMSCs after osteogenic induction, but BMSCs treated with either of these three EGM‐EVs had less mineralized nodule formation relative to the vehicle control group (Figure 5I,J). The significant pro‐osteogenic effect of these three CGM‐EVs and *Akk*‐EVs was further confirmed by qRT‐PCR analysis for the expression of genes related to osteogenesis (*Bglap*, *Alpl*, and *Runx2*) in BMSCs under osteogenic induction (Figure S7D–F, Supporting Information). TRAP staining showed that these three CGM‐EVs and *Akk*‐EVs markedly reduced the number and size of osteoclasts formed by RAW264.7 cells, but all EGM‐EVs resulted in further increases of osteoclast number and size (Figure 5K,L; Figure S7G, Supporting Information). Consistently, qRT‐PCR analysis for the expression of osteoclastogenesis‐related genes (*Trap*, *Ctsk*, *Mmp9*, *Oc‐stamp*, and *Oscar*) also determined the anti‐osteoclastogenic action of all CGM‐EVs and *Akk*‐EVs, and the pro‐osteoclastogenic effect of all EGM‐EVs (Figure S7H–L, Supporting Information). EGM‐EVs combined with *Akk*‐EVs also promoted mineralized nodule formation of BMSCs and reduced the number and size of osteoclasts formed by RAW264.7 cells, even though the effects were much lower than *Akk*‐EVs (Figure 5I–L; Figure S7G, Supporting Information), suggesting that EGM‐EVs + *Akk*‐EVs or EGM + *Akk* may have the potential to induce bone protective effect in mice. The non‐EV‐containing fractions from the culture supernatant of different donor‐derived CGM, different donor‐derived EGM, or *Akk* did not induce significant effects on calcium nodule formation of BMSCs, as well as on the number and size of osteoclasts formed by RAW264.7 cells (Figure 5I–L; Figure S7G, Supporting Information). These results suggest that the secreted EVs, but not other paracrine factors, primarily contribute to the pro‐osteogenic and anti‐osteoclastic effects of CGM and *Akk*. *E. coli*‐EVs significantly impaired osteogenesis of BMSCs and inhibited osteoclast differentiation of RAW264.7 cells, but the anti‐osteoclastic effect was much lower compared with CGM‐EVs and *Akk*‐EVs (Figure 5I–L; Figure S7G, Supporting Information). The regulatory properties of these bacterial EVs on osteogenesis and osteoclastogenesis suggest their potential as modulators to directly regulate bone homeostasis.

### 2.7 CGM‐EVs and *Akk*‐EVs Protect against OVX‐Induced Osteoporosis

**Figure 6:**
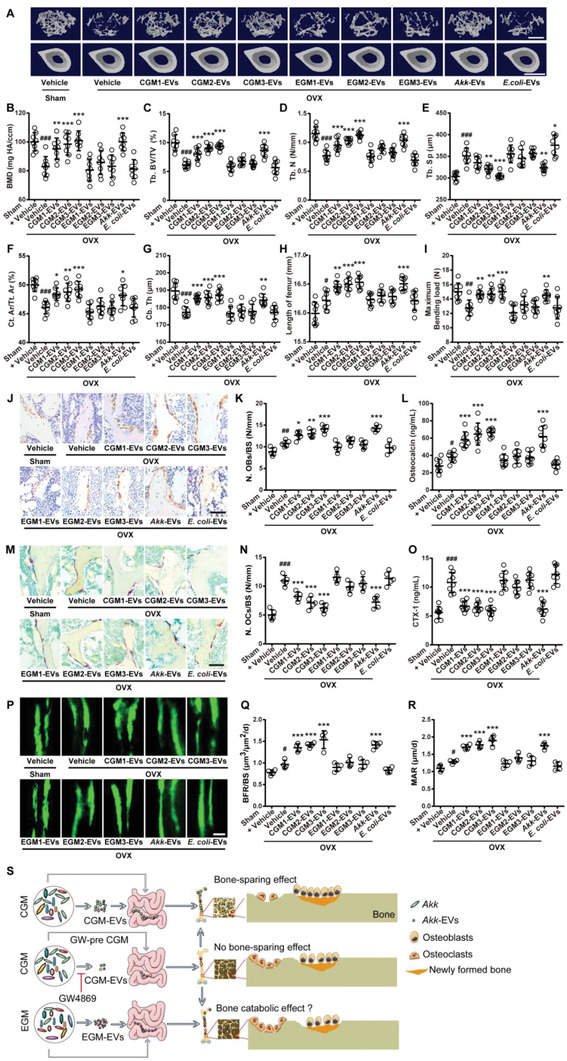
CGM‐EVs and *Akk*‐EVs protect against OVX‐induced osteoporosis after oral administration. A) Representative µCT images of trabecular (up) and cortical (bottom) bone in femora from Sham and OVX mice receiving vehicle or different EVs by oral route. Scale bars: 500 µm (up) and 1 mm (bottom). Quantification of B) BMD, C) Tb. BV/TV, D) Tb. N, E) Tb. Sp, F) Ct. Ar/Tt. Ar, and G) Ct. Th. *n* = 8 per group. H) Lengths of femora. *n* = 8 per group. I) Femur ultimate load measured by three‐point bending test. *n* = 8 per group. J) Representative OCN‐stained sections with quantification of K) osteoblast number. Scale bar: 50 µm. *n* = 5 per group. L) ELISA for serum OCN. *n* = 8 per group. M) Representative TRAP‐stained sections with quantification of N) osteoclast number. Scale bar: 50 µm. *n* = 5 per group. O) ELISA for serum CTX‐I. *n* = 8 per group. P) Representative images of calcein double labeling with quantitation of Q) BFR/BS and R) MAR. Scale bar: 10 µm. *n* = 4 per group. S) Schematic diagram summarizing the major findings of this study. Data are presented as mean ± SD. **^#^** *P* < 0.05 versus Sham + Vehicle group, * *P* < 0.05 versus OVX + Vehicle group. ^#/*^ *P* < 0.05, ^##/**^ *P* < 0.01, and ^###/***^ *P* < 0.001.

We then examined whether direct treatment with CGM‐EVs from different donors (CGM1‐EVs, CGM2‐EVs, and CGM3‐EVs) and *Akk*‐EVs at the dose of 1.2 × 10^10^ vesicles (≈200 µg protein contents) twice a week for 2 months is sufficient to protect the OVX mice from osteoporosis, and compared their effects with EGM‐EVs from different donors (EGM1‐EVs, EGM2‐EVs, and EGM3‐EVs) and *E. coli*‐EVs. µCT, femur length analysis, and three‐point bending test, respectively, showed that the oral administration of CGM‐EVs from different donors or *Akk*‐EVs profoundly reversed the decrease of trabecular and cortical bone mass, improved bone microarchitecture, increased femur length, and enhanced bone strength in OVX mice (**Figure** **6**A–I; Figure S8A–C, Supporting Information). However, the OVX mice treated with EGM‐EVs or *E. coli*‐EVs still had similar levels of BMD, bone microstructural parameters, bone length and bone strength compared with the vehicle‐treated OVX mice (Figure 6A–I; Figure S8A–C, Supporting Information). The oral treatment of CGM‐EVs and *Akk*‐EVs could also increase osteoblast number and osteogenic activity in OVX mice, as revealed by OCN immunostaining (Figure 6J,K) and ELISA for serum OCN (Figure 6L), respectively. TRAP staining (Figure 6M,N; Figure S8D, Supporting Information) and ELISA test for CTX‐I (Figure 6O) revealed that CGM‐EVs and *Akk*‐EVs inhibited the OVX‐induced increases in the number and size of osteoclasts as well as bone resorption activity. Calcein double labeling demonstrated profoundly increased new bone formation and mineralization in OVX mice after oral administration of CGM‐EVs or *Akk*‐EVs (Figure 6P–R). Although our *in vitro* results showed that EGM‐EVs could inhibit osteogenesis and increase osteoclastogenesis, and *E. coli*‐EVs exerted an inhibitory role in these two processes, these vesicles at the current dose did not induce obvious impacts on osteoblastic bone formation and osteoclastic bone resorption in OVX mice (Figures 5I–L and 6J–R). The beneficial effects on bone mass, bone microarchitecture, and bone length were also observed in OVX mice intravenously injected with CGM‐EVs from different donors or *Akk*‐EVs, but not in those treated with EGM‐EVs, as indicated by the µCT scanning data and the changes of femur length values (Figure S9A–K, Supporting Information). Collectively, these results indicate that CGM‐EVs and *Akk*‐EVs, like CGM and *Akk*, are capable of preserving bone mass and bone strength by promoting bone formation and inhibiting bone resorption. With the aging of GM donor, the bone protective effect of GM‐derived EVs is lost. These findings further suggest the mediatory role of EVs in the function of GM.

Taken together, our findings suggest that EVs from CGM, especially from *Akk* in CGM, exert bone‐sparing effects by promotion of osteoblastic bone formation and suppression of osteoclastic bone resorption (Figure 6S). EGM lacks *Akk* and administration of EGM or its EVs cannot induce bone protective effects; although EGM or EGM‐EVs at the current dose do not cause notable bad effects on osteoporotic bone, EGM‐EVs can decrease osteogenesis and increase osteoclast formation *in vitro*, indicating a catabolic effect of EGM on bone (Figure 6S).

## 3 Discussion

Bone has long been linked with the gastrointestinal tract system due to the requirement for calcium absorption to facilitate bone mineralization, but additional factors in the gut such as GM and its metabolites, immune cells, cytokines, incretins, and serotonin also contribute to the regulation of bone health.^[^ ^31^ ^]^ Studies conducted to date offer substantial evidences that a GM‐bone axis exists.^[^ ^2^, ^4^, ^5^, ^6^, ^7^, ^31^, ^32^ ^]^ Our study has three major findings that enrich the knowledge of GM‐bone regulatory axis. First, we confirm the beneficial role of GM from children (CGM) , but not from elderly donors (EGM), in protecting against OVX‐induced osteoporosis. Second, we identify that a bacterial species in GM, *Akk*, is beneficial for the maintenance of bone mass and strength, and likely a critical mediator of the CGM‐induced anti‐osteoporotic effects. Third, we present a novel communication mode between GM and bone through the long‐distance transmission of functional EVs from the bacteria to bone cells. Our study suggests that the *Akk*‐abundant CGM restores the OVX‐induced loss of *Akk*, which produces bone‐beneficial EVs and finally enhances bone mass and strength in OVX mice.

The loss of estrogen due to menopause is the major risk factor for osteoporosis. Recent evidence by Li et al. reported that estrogen deprivation induced by leuprolide (a gonadotrophin releasing hormone agonist) fails to augment osteoclastic bone resorption and induce trabecular bone loss in germ‐free mice, whereas the germ‐free mice recolonized with GM and the conventionally raised control mice are shown to lose bone following leuprolide treatment,^[^ ^33^ ^]^ suggesting that GM is essential for the development of estrogen deficiency‐induced osteoporosis. Herein, we found that estrogen deficiency induced by OVX resulted in shifts of GM composition, especially led to a marked decrease of *Akk*; modulating the gut bacteria by colonization with the exogenous *Akk*‐abundant CGM or by direct replenishment of *Akk* effectively reversed the OVX‐induced imbalanced bone metabolism and enhanced trabecular and cortical bone mass, bone length, and bone strength in mice. However, treatment with EGM, which had low level of *Akk*, did not evoke any bone protective effects in OVX mice. These findings suggest that the loss of *Akk* may be an important factor contributing to the pathogenesis of postmenopausal osteoporosis and the restoring of *Akk* abundance is an important mechanism by which CGM benefits bone. The administration of either the *Akk*‐abundant CGM or pure *Akk* appears to be a promising strategy for beneficially modulating the host gut microbiome to improve bone health.

Previous studies have reported that *Akk* abundance is reduced in aged humans and mice. Collado et al. reported that the levels of *Akk* in fecal samples from elderly subjects (80 to 82 years old) are decreased compared to healthy adults (25 to 35 years old) in Finland.^[^ ^34^ ^]^ Wang et al. showed that *Akk* abundance is lower in Chinese centenarians (100–108 years old) than that in Chinese younger elderly (85–99 years old).^[^ ^35^ ^]^ A significant decline of *Akk* with aging is also observed in mice.^[^ ^36^, ^37^ ^]^ Consistently, in our study, we found that *Akk* abundance was higher in fecal samples of 3 to 5 years old CGM donors compared with 64 to 78 years old EGM donors from China. Considering the role of aging in the pathogenesis of osteoporosis and the different effects of CGM and EGM on bone, the loss of *Akk* might also contribute to the development of senile osteoporosis, which warrants future investigation. However, there exists evidence that *Akk* abundance is increased in healthy adults (25 to 35 years old) relative to healthy infant subjects (1‐, 6‐, or 12‐month‐old).^[^ ^34^ ^]^ Biagi et al. reported higher levels of *Akk* in Italian elderly (60 to 80 years old) and centenarians than those in Italian young adults (20 to 40 years old).^[^ ^38^ ^]^ The differences in the ages of the donors in different studies may contribute to the discrepancies of the changes of *Akk* abundance with aging. Other factors, such as the differences in the donors’ races, regions, lifestyles, dietary habits, body weights, and metabolic status, may also cause the discrepancies of the results. In our study, although various parameters revealing the metabolic status were within the normal range in all EGM donors, there seemed to be a trend toward unhealthy metabolic status in EGM donors compared to CGM donors, consistent with previous studies that aging is accompanied by changes in metabolic biochemical indicators.^[^ ^39^ ^]^ Thus, the metabolic alterations in EGM donors may be associated with the reduction of *Akk*, which still needs further exploration.

A phenomenon should be noted that although transplantation with EGM and its EVs at the current dose just caused a trend of increase in serum level of bone resorption marker CTX‐I in OVX mice, treatment with EGM‐EVs markedly impaired osteogenesis and enhanced osteoclast formation *in vitro*. These findings, along with some evidences that GM has detrimental effects on bone mass,^[^ ^2^, ^6^, ^7^ ^]^ suggest the existence of gut microbes that are harmful to bone health in EGM and may play an important role in the etiology of senile osteoporosis, which requires future investigation.

Probiotics are defined as “live microorganisms that when administered in adequate amounts will confer a health benefit on the host.”^[^ ^40^ ^]^ Treatments with probiotics, such as *Lactobacillus reuteri*,^[^ ^9^ ^]^ *Lactobacillus paracasei*,^[^ ^41^ ^]^ *Lactobacillus plantarum*,^[^ ^4^, ^41^ ^]^ *Lactobacillus rhamnosus GG*,^[^ ^33^ ^]^ and *Bifidobacterium longum*,^[^ ^42^ ^]^ have been shown to beneficially modulate bone health. *Akk* is a Gram‐negative, anaerobic mucin‐degrading bacterium that belongs to the phylum *Verrucomicrobia* and resides in the gastrointestinal tract of human and animals.^[^ ^23^, ^43^ ^]^ Since its discovery by Derrien et al.,^[^ ^23^ ^]^ *Akk* has increasingly been studied and recognized as a new functional microbe with probiotic properties. This bacterium is present in high levels (1–5%) in the intestinal tract of humans and reduced during a variety of diseases including obesity, metabolic syndrome, diabetes, colitis, and psoriasis.^[^ ^44^, ^45^, ^46^ ^]^ Higher *Akk* abundance is associated with a healthier metabolic status in obese individuals undergoing caloric restriction.^[^ ^47^ ^]^ Loss of *Akk* impairs intestinal integrity and increases gut leakiness, which ultimately results in metabolic endotoxemia, inflammation, and insulin resistance.^[^ ^36^ ^]^ *Akk* treatment by oral gavage is able to reverse high‐fat diet‐induced obesity, metabolic endotoxemia, adipose tissue inflammation, and insulin resistance.^[^ ^45^ ^]^ A recent study by Plovier et al. showed that the pasteurized *Akk* or *Akk*‐derived outer membrane protein Amuc_1100 can improve the gut barrier and protect mice from dietary‐induced fat‐mass gain and insulin resistance.^[^ ^46^ ^]^ Li et al. reported that *Akk* has the ability to prevent western diet‐induced atherosclerosis by inhibiting endotoxemia‐induced inflammation in *Apoe* ^−/−^ mice.^[^ ^48^ ^]^ Herein, we provided the first evidence that the presence of gut bacterium *Akk* was positively correlated with bone mass, length, and strength parameters. Therapeutic intervention targeting this single species was sufficient to correct the OVX‐induced imbalance between bone resorption and bone formation and protect mice from osteoporosis. Our findings suggest the prospect of *Akk* as a probiotic bacterium for the prevention and/or treatment of osteoporosis. More recently, Lawenius et al. reported that the oral administration of pasteurized *Akk* every day for 4 weeks reduces bone mass in normal mice and cannot protect against bone loss in OVX mice.^[^ ^27^ ^]^ Two reasons may account for the different outcomes of *Akk* transplantation on bone health between our study and the study by Lawenius et al. The first reason is likely that the process of pasteurization causes *Akk* death and impairs the production of functional *Akk*‐EVs. The negative effect of pasteurized *Akk* on bone mass in normal mice^[^ ^27^ ^]^ may be due to that the death of *Akk* results in the generation of some substances detrimental to bone, which still needs future investigation. Second, the difference in the frequency of *Akk* treatment may also lead to two opposite outcomes. In our study, *Akk* transplantation was conducted twice a week for 8 weeks. However, *Akk* treatment in study by Lawenius et al. was performed every day for 4 weeks.^[^ ^27^ ^]^ Very high frequency of *Akk* treatments may be detrimental to bone. In future studies, it is worthwhile to compare the effects of *Akk* transplantation on bone mass at different treatment frequencies.

The mechanisms through which GM exerts regulatory effects on bone mass are still being investigated. Several possible mechanisms have been reported to underlie the relationship between GM and bone metabolism. GM may induce enterochromaffin cells to produce serotonin, a molecule that has negative effects on bone formation by inhibiting osteoblast proliferation.^[^ ^2^, ^32^, ^49^, ^50^ ^]^ Some bacteria have been shown to synthesize vitamins like D, C, K, and folate, which are required for matrix formation and bone growth.^[^ ^8^, ^51^ ^]^ Bacteria can also produce short chain fatty acids (SCFAs), which are thought to reduce the intestinal tract pH and subsequently increase calcium absorption.^[^ ^31^ ^]^ In 2018, Tyagi et al. reported that *L. rhamnosus GG* increases the serum levels of the SCFA butyrate and thereby stimulates bone formation through the regulatory T cells‐mediated regulation of WNT10B expression.^[^ ^52^ ^]^ Yan et al. showed that GM has the ability to enhance the levels of serum insulin‐like growth factor 1 (IGF‐1) and increase bone formation by promoting the production of SCFAs.^[^ ^5^ ^]^ Another possible mechanism by which GM affects bone health is modulating the immune system and the expression of pro‐inflammatory and pro‐osteoclastogenic cytokines.^[^ ^9^, ^33^, ^50^, ^53^ ^]^ A study by Britton et al. indicated that the *L. reuteri*‐induced bone protective effects in OVX mice may be associated with the suppression of CD4^+^ T cells and production of pro‐osteoclastogenic factors.^[^ ^53^ ^]^

Bacterial EVs carry various cargoes from their parent bacteria (such as lipopolysaccharide, peptidoglycan, nucleic acids, and proteins) and serve as a vehicle to deliver these molecules into host cells, thereby regulating the biological activities of the recipient cells.^[^ ^15^, ^16^, ^54^ ^]^ Bacterial EVs may also incorporate cell membrane‐associated compounds beneficial to host health, such as vitamin K2 and certain fatty acids.^[^ ^55^ ^]^ In our study, we present a novel mechanism underlying the exchange of signals between the microbiota and the host bone through the transfer of functional EVs from the bacteria to bone cells. We found that EVs secretion was required for CGM‐induced anti‐osteoporotic effects and the direct oral administration of CGM‐EVs or *Akk*‐EVs could efficiently enhance bone mass and strength in OVX‐induced osteoporotic mice. Results of tracking experiments confirmed the capacity of these EVs to accumulate into the mouse bone tissues. These findings, along with the evidence that these EVs directly promoted osteogenesis and suppressed osteoclastogenesis *in vitro*, suggest that GM can communicate with the host bone cells through the secretion of EVs, which penetrate through the host intestinal barriers, enter the systemic circulation, and then are transported into bone tissues to directly regulate osteoblastic bone formation and osteoclastic bone resorption. CGM‐EVs and *Akk*‐EVs may contain some molecules that exert pro‐osteogenic or/and anti‐osteoclastogenic function and contribute to the beneficial effects of these EVs on bone health. The components that mediate the bone‐protective function of these EVs still require future investigation.

## 4 Experimental Section

##### Collection of GM

Fecal samples (the elderly: ≈100–150 g per sample; children: about 50–75 g per sample) for preparing CGM and EGM samples, respectively, were collected from three healthy children (two boys and one girl; 3–5 years old) and three healthy old people (two men and one woman; 64–78 years old) from three different families (one CGM donor and one EGM donor per family). The CGM donor and EGM donor in each family lived together and often had dinner together. No CGM or EGM donor in each family was found to have a unique diet. Fecal samples for assessing the changes of microbial composition and evaluating the abundance of *Akk* between CGM and EGM were also collected from the above described donors. Inclusion criteria were as follows: no severe chronic disease (hypertension, cardiovascular disease, diabetes, tumor, etc.), no infectious disease, and no use of antibiotics during past three months. Written informed consents were obtained from all fecal donors or their parents and all experimental procedures were approved by the Ethical Review Board at Xiangya Hospital of Central South University. For GM preparation, once collected, each fecal sample was shipped to laboratory on ice immediately as described previously.^[^ ^56^ ^]^ Briefly, fecal sample was homogenized with reduced sterilized normal saline (normal saline supplemented with 0.05% l‐cysteine‐HCl) at ratio of 1:5. For removing undigested food and smaller particles, slurry was passed through 2.0, 1.0, 0.5, and 0.25 mm pore size stainless steel sieves and then centrifuged at 6000 × *g* for 15 min at 4 °C. After removing the supernatant, GM was resuspended in half of the original volume reduced sterilized normal saline and then added with sterile glycerol to a final concentration of 10%. The whole procedure should be performed anaerobically and finished within 1 hour. Small amount of each prepared GM solution was subjected to agar plating to assess bacteria concentration. To evaluate the effects of each of CGM and EGM from the above‐described donors on bone, the obtained each CGM or EGM sample was directly divided into small aliquots and used immediately for the downstream experiments or stored at −80 °C (≤8 weeks) before use. To assess the impacts of the pooled CGM or EGM from different donors on bone, GM preparation from three CGM or EGM donors was mixed with equal amount and used immediately or stored in small aliquots at −80 °C (≤8 weeks) before use.

##### Culture of Bacteria

*Akk* (ATCC BAA‐835, Manassas, VA) was cultured in BHI broth (BD Bioscience, San Jose, CA, USA) containing 0.05% l‐cysteine‐HCl (Sigma‐Aldrich, St Louis, MO, USA) and 0.5% porcine mucin (Sigma‐Aldrich) as described previously.^[^ ^48^ ^]^ *E. coli* ATCC25922 was grown at 37 °C in LB medium (MB2454, Meilunbio, Guangzhou, China). Bacteria were shaken at 170 r.p.m. and incubated under anaerobic (for *Akk*) or aerobic (for *E. coli*) condition at 37 °C. The concentration of bacteria was calculated by measuring the absorbance at the wavelength of 600 nm^[^ ^48^, ^57^ ^]^ or by counting the numbers of colony forming units (CFUs) of bacteria after plating on BHI or LB agar with the corresponding supplements.

##### Isolation and Characterization of Bacterial EVs

For isolation of CGM‐EVs and EGM‐EVs, CGM and EGM at the concentrations of 1 × 10^7^ CFUs per 100 mL medium were cultured in YCFA medium (1 g casitone, 0.25 g yeast extract, 0.4 g NaHCO_3_, 0.1 g cysteine, 0.045 g K_2_HPO_4_, 0.045 g KH_2_PO_4_, 0.09 g NaCl, 0.009 g MgSO_4_·7H_2_O, 0.009 g CaCl_2_, 0.1 mg resazurin, 1 mg haemin, 1 µg biotin, 1 µg cobalamin, 3 µg *p*‐aminobenzoic acid, 5 µg folic acid, and 15 µg pyridoxamine) supplemented with 0.002 g mL^−1^ each of glucose, maltose, and cellobiose under anaerobic condition^[^ ^58^ ^]^ for 4 days. For isolation of *Akk*‐EVs and *E. coli*‐EVs, *Akk* and *E. coli* (1 × 10^7^ CFUs per 100 mL medium) were cultured in BHI or LB broth with the above‐described supplements for 4 days. Then, the conditioned media of these bacteria were obtained, centrifugated at 6000 × *g* for 30 min at 4 °C, and then filtered through a 0.22 µm filter (Millipore, Billerica, USA) to remove the residual bacteria. The supernatant was transferred to Amicon Ultra‐15 Centrifugal Filter Units (100 kDa; Millipore) and concentrated 400‐ to 500‐fold by centrifugation at 4000 × *g* and 4 °C. EVs were then isolated from the ultrafiltration liquid by bottom‐up Optiprep density gradient centrifugation. Briefly, 1.33 mL of bacterial EVs were mixed with 6.67 mL Optiprep solution (60% w/v iodixanol; Sigma Aldrich) to generate a 50% layer in a 38.5 mL polyallomer Beckman Coulter tube. 8 mL of 40%, 8 mL of 20%, and 7 mL of 10% Optiprep and 2 mL PBS were sequentially layered on the top of the 50% layer. The Optiprep density gradient (ODG) fractions were then centrifuged at 100 000 × *g* with a Beckman Optima XPN ultracentrifuge (SW 32 Ti rotor; *k*‐factor 204) for 18 h at 4 °C. The ODG fractions of 2 mL each (the bottom fraction was about 1 mL) were collected and DLS results for each fraction enabled the selection of the EV‐rich fractions (fractions 8–9). The EV‐rich fractions were diluted to 30 mL with PBS and centrifuged at 100 000 × *g* for 3 h at 4 °C to obtain EVs pellet. The non‐EV‐containing fractions served as controls. Agar plating was performed to ensure no bacterial contamination in the purified EVs.^[^ ^15^ ^]^ The obtained EVs were used immediately or stored at −80 °C until use (avoiding multiple frozen‐thaw cycles). The protein contents of EV samples and non‐EV‐containing fractions were assessed by Pierce BCA protein assay kit (Thermo Fisher Scientific, Waltham, USA). The numbers of EVs were tested by nanoparticle tracking analysis. The morphologies of EVs were detected by a Hitachi H‐7650 transmission electron microscope (Hitachi, Tokyo, Japan) and the sizes of EVs were tested by DLS using a Nanosizer instrument (Malvern Instruments, Malvern, UK).

##### Inhibition of EV Secretion

To confirm whether GW4869 is capable of inhibiting EV secretion by CGM or *Akk*, CGM or *Akk* (10^7^ CFUs per 100 mL medium) was cultured in complete medium containing GW4869 (10 × 10^−6^ m; Santa Cruz Biotechnology, Santa Cruz, USA) or an equal volume of vehicle (DMSO). Bacteria grew under anaerobic condition and were shaken at 170 r.p.m. and 37 °C. 4 days later, the viability of CGM and *Akk* was assessed by bacterial colony counting assay on YCFA or BHI agar plates with the corresponding supplements as described above. The conditioned media of CGM and *Akk* were harvested for the isolation of CGM‐EVs and *Akk*‐EVs by Optiprep density gradient centrifugation. The CGM and *Akk* pellets were respectively resuspended in fresh complete medium and continued to grow for another 4 days. The conditioned media were obtained for the isolation of CGM‐EVs and *Akk*‐EVs, in order to evaluate the lasting inhibitory effects of GW4869 on EV secretion by CGM and *Akk*. The protein content and particle number of the isolated EVs were measured using a BCA Protein Assay Kit and nanoparticle tracking analysis, respectively.

##### Animal Experiments

12‐week‐old C57BL/6 female mice were used in this study. Animal care and experimental procedures were approved by the Ethical Review Board at Xiangya Hospital of Central South University. The mice were generally anesthetized and subjected to either a sham operation (Sham) or bilateral OVX as described previously.^[^ ^22^ ^]^ To assess the effects of different types of bacteria on postmenopausal osteoporosis, the OVX mice were orally administered with 7.5 × 10^9^ CFUs of pooled CGM, pooled EGM, or unmixed CGM or EGM from different donors, or with 3 × 10^8^ CFUs of *Akk* or *E. coli*. To investigate whether EV secretion is required for the CGM‐ or *Akk*‐induced regulation of bone health, the OVX mice were orally administered with 7.5 × 10^9^ CFUs of GW4869‐pretreated CGM or DMSO (the vehicle of GW4869)‐pretreated CGM, or with 3 × 10^8^ CFUs of GW4869‐pretreated *Akk* or DMSO‐pretreated *Akk*. To evaluate the effects of EVs on OVX‐induced osteoporosis, the OVX mice were orally administered with 1.2 × 10^10^ vesicles of different donor‐derived CGM‐EVs (CGM1‐EVs, CGM2‐EVs, and CGM3‐EVs), different donor‐derived EGM‐EVs (EGM1‐EVs, EGM2‐EVs, and EGM3‐EVs), *Akk*‐EVs, or *E. coli*‐EVs in 0.75 mL PBS, or were intravenously injected with 3 × 10^9^ vesicles of different donor‐derived CGM‐EVs (CGM1‐EVs, CGM2‐EVs, and CGM3‐EVs), different donor‐derived EGM‐EVs (EGM1‐EVs, EGM2‐EVs, and EGM3‐EVs), or *Akk*‐EVs in 200 µL PBS through the tail vein. The mice in Sham + Vehicle group and OVX + Vehicle group were treated with an equal volume of vehicle of bacteria or EVs. The above treatments were conducted twice a week for 2 months. At the age of 5 months, fecal samples from the vehicle‐treated Sham mice and the CGM‐, EGM‐ or vehicle‐treated OVX mice were collected and stored at −80 °C before 16S rRNA gene sequencing or qRT‐PCR analysis for 16S rRNA gene of *Akk*. All mice were weighed, anesthetized, taken blood by enucleation of the eyeball, and then sacrificed. Serum samples were obtained by centrifugation at 1000 × *g* and 4 °C for 15 min and then stored at −80 °C before analyses. Femora were harvested for the downstream measurements. Uteri were isolated and weighed to verify the success of OVX procedure.

##### µCT Analysis

Femora dissected from mice were fixed overnight in 4% paraformaldehyde and analyzed by high‐resolution μCT (VIVACT 80; SCANCO Medical AG, Switzerland) as described in the previous studies.^[^ ^21^, ^22^, ^59^ ^]^ The scanner was set at a current of 200 µA and a voltage of 70 kV, respectively. The isotropic voxel size, X‐ray tube potential and integration time were set at 11.4 × 11.4 × 11.4 µm^3^, 55 kVp, and 400 ms, respectively. The image reconstruction software (NRecon), data analysis software (CTAn v1.11), and 3D model visualization software (μCTVol v2.2) were applied to analyze the parameters of the distal femoral metaphyseal trabecular bone and the diaphyseal cortical bone. Region of interest (ROI) selected for trabecular bone analysis was started from 0.3 mm proximal to the distal growth plate and extended proximally for 5% of femoral length, which excluded the growth plate and primary spongiosa. The fixed threshold values were set to 60 as the lower limit and 255 as the upper limit to distinguish mineralized bone from other tissues. BMD, Tb. BV/TV, Tb. N, Tb. Th, and Tb. Sp were measured. For cortical bone, the ROI selected for scanning was started from 40% of femoral length proximal to the distal growth plate and extended proximally for 10% of femoral length, by which Ct. Ar/Tt. Ar, Ct. Ar, Tt. Ar, and Ct. Th were calculated.

##### Biomechanical Test

Three‐point bending test was performed to evaluate the mechanical properties of femora using a universal testing machine (Instron 3343; Instron, Canton, USA).^[^ ^60^ ^]^ Briefly, femora were placed in the anterior–posterior direction (patella side facing up) on the lower supporting bars at 8 mm apart. A constant vertical compression load (5 mm min^−1^) was exerted to the midpoint of the samples until fracture happened. The maximum bending load of femur (N) was calculated from load displacement curve.

##### Histological, Immunohistochemical, and Histomorphometric analyses

For histological and immunohistochemical staining, femora were fixed in 4% paraformaldehyde for 48 h and decalcified in 0.5 m EDTA (pH = 7.4) at 4 °C with continuous shaking for about 3 days. After that, the samples were dehydrated in a graded series of ethanol and embedded in paraffin. Bone samples were sectioned longitudinally into 5 µm thick slices and stained for OCN and TRAP as described previously,^[^ ^21^, ^22^, ^59^ ^]^ in order to detect OCN^+^ osteoblasts and TRAP^+^ osteoclasts within the entire ROI that was also selected for trabecular bone μCT analysis. The endocortical surface and periosteal surface of the cortical bone were not included in the analysis. Images were acquired with an Olympus CX31 optical microscope (Olympus, Tokyo, Japan). The numbers of osteoblasts or osteoclasts on secondary spongiosa cancellous bone surface in the entire ROI were counted and the data were then normalized to the number of osteoblasts or osteoclasts per millimeter of trabecular bone surface perimeter (N mm^−1^). The size of osteoclasts on secondary spongiosa cancellous bone surface was evaluated by assessing osteoclast perimeter (OCs PM) per millimeter of trabecular bone perimeter (Tb PM) within the entire ROI. OCN primary antibody (Cat. No gb11233; 1:200) and its secondary antibody (Cat. No gb23303; 1:200) were purchased from Servicebio (Wuhan, China). TRAP staining kit (Cat. No 387A‐1KT) was purchased from Sigma‐Aldrich.

To determine dynamic bone formation, 0.1% calcein (Sigma‐Aldrich; 10 mg kg^−1^ body weight) in PBS was intraperitoneally injected into the mice at 9 and 3 days before sacrifice. Then, tibias were collected and fixed in 4% paraformaldehyde for 48 h, dehydrated in increasing concentrations of ethanol, and embedded in methyl methacrylate. 10 µm thick sections of the undecalcified bones were prepared and calcein double labeling was observed under a fluorescence microscope (Leica DMI6000B, Solms, Germany). The ROI selected for analysis was started from 0.3 mm distal to the proximal epiphyseal growth plate and extended distally for 20% of tibial length, which included the secondary spongiosa cancellous bone and excluded the growth plate and primary spongiosa. The endocortical surface and periosteal surface of the cortical bone were not included in the analysis. BFR/BS and MAR were measured within the entire ROI by using Image‐Pro Plus 6 software.

##### ELISA

The concentrations of serum OCN and CTX‐I proteins were measured using commercial ELISA kits (Elabscience, Wuhan, China). All the procedures were performed in accordance with the manufacturer's instruction.

##### Preparation of Antibody Targeting *Akk*‐EVs

The immunization of rabbits with purified *Akk*‐EVs and the preparation of polyclonal antibody targeting *Akk*‐EVs were performed by FriendBio Technology (Wuhan, China). Briefly, after collecting preimmune serum (≥1 mL per rabbit), two 12‐week‐old New Zealand male rabbits were immunized with *Akk*‐EVs (3 × 10^10^ vesicles per rabbit) by subcutaneous injection. Each rabbit was boosted twice with *Akk*‐EVs (3 × 10^10^ vesicles per subcutaneous injection) at 10 and 20 days after the priming dose. The serum antibody titer was tested by ELISA at days 27 after first immunization. One week later, the rabbits were boosted for the third time with *Akk*‐EVs (100 µg per rabbit. ≈ 6 × 10^10^ vesicles ) through the ear vein. 10 days later, rabbits were bled and their serum was collected and kept at −20 °C before use. The antibody titer (displayed in Table S4 in the Supporting Information) was assayed by ELISA and the serum antibodies showing a higher titer against *Akk*‐EVs were chosen for downstream experiment.

##### *In Vivo* Tissue Distribution of EVs

Different donor‐derived CGM‐EVs (CGM1‐EVs, CGM2‐EVs, and CGM3‐EVs), different donor‐derived EGM‐EVs (EGM1‐EVs, EGM2‐EVs, and EGM3‐EVs), or *Akk*‐EVs were labeled with a lipophilic dye DIR iodide (Santa Cruz) according to the manufacturer's instruction for *ex vivo* fluorescent imaging. Bottom‐up Optiprep density gradient centrifugation with the procedures described in detail as above was performed to remove the possible redundant fluorescent dye in the DIR‐labeled CGM‐EVs, EGM‐EVs or *Akk*‐EVs. The obtained EV‐rich fractions were diluted to 30 mL with PBS and centrifuged for 3 h at 100 000 × *g* and 4 °C. The EV pellets were resuspended in PBS and administered by different routes (oral: 1.2 × 10^10^ EVs in 100 µL PBS; rectal: 1.2 × 10^10^ EVs in 100 µL PBS; intravenous: 3 × 10^9^ EVs in 100 µL PBS) to each mouse that had been fasted 48 h. The mice in control group were treated with an equal volume of vehicle. 1 h later, all mice were sacrificed to collect femora, tibias, muscles (quadriceps femoris muscle and triceps surae muscle), livers, kidneys, and brains. After fixing in 4% paraformaldehyde for 15 min, fluorescent signals in these organs were detected and quantified with a fluorescence tomography imaging system (FMT‐4000; PerkinElmer, USA).

The above‐mentioned three CGM‐EVs, three EGM‐EVs, and *Akk*‐EVs were also labeled with a green fluorescent dye (PKH67; Cat. No MINI67; Sigma‐Aldrich) for testing the tissue distribution of these EVs. After removing the redundant dye by procedures described above, 100 µL PBS with or without 1.2 × 10^10^ vesicles of CGM‐EVs, EGM‐EVs or *Akk*‐EVs were orally administrated to the fasted mice. 1 h later, the femur, brain, kidney, and liver tissues were collected and fixed in ice‐cold 4% paraformaldehyde for 4 h. Femora were decalcified in 0.5 m EDTA (pH = 7.4) at 4 °C with constant shaking for about 3 days. All samples were immersed into 30% sucrose overnight for dehydration. After exposure to liquid nitrogen for a few seconds, samples were embedded in optimal cutting temperature (OCT) compound (Sakura Finetek USA, Inc., Torrance, CA, USA) and sectioned into 5 µm thick slices. Sections were rinsed in three changes of PBS for 5 min each and DAPI (0.5 µg mL^−1^; Invitrogen, Carlsbad, USA) was then used to stain nuclei. Images were obtained with a confocal microscope (Leica SP8, Mannheim, Germany) and the mean intensity of fluorescent signals was measured by Image J 1.51j8.

To detect the biodistribution of *Akk*‐EVs using serum containing antibodies targeting *Akk*‐EVs, the fasted mice were orally treated with 1.2 × 10^10^ vesicles of *Akk*‐EVs or an equal volume of vehicle (100 µL PBS) for 1 h. After killing the mice, the femur, brain, kidney, and liver tissues were harvested, followed by being processed to 5 µm thick sections by procedures described above. The sections were blocked for 0.5 h with 3% donkey serum and then incubated overnight at 4 °C with serum from rabbit immunized with *Akk*‐EVs. The sections incubated with preimmune serum from the rabbit served as negative control group. After washing, the sections were immunoreacted with the secondary antibody Alexa Fluor 488 AffiniPure Donkey Anti‐Rabbit IgG (H+L) (Cat. No. 711‐545‐152; Jackson ImmunoResearch; 1:300). DAPI was used to stain nuclei. Images were obtained and the mean intensity for the positively stained areas was assessed by Image J 1.51j8.

##### Cell Culture

Primary BMSCs were isolated from the femurs and tibias of the wild‐type C57BL/6 mice as described in previous studies by our group and others.^[^ ^61^ ^]^ BMSCs were cultured in *α*‐MEM (Cat. No. SH30265.01; Hyclone, Logan, USA) containing 10% fetal bovine serum (FBS; Cat. No. 12664‐025; Gibco, Grand Island, USA) and 1% penicillin–streptomycin (PS; Cat. No. P1400; Solarbio, Beijing, China). The murine osteoclast progenitor RAW264.7 cells were obtained from American Type Culture Collection (Rockville, MD, USA) and incubated in high glucose DMEM (Gibco) supplemented with 10% FBS and 1% PS. Cell were cultured at 37 °C and 5% CO_2_ in a humidified atmosphere.

##### EVs Uptake Assay

EVs were labeled with PKH26 (Cat. No MINI26; Sigma‐Aldrich) according to manufacturer's instructions. After removing the redundant dye by procedures described above, the labeled EVs (6 × 10^8^ vesicles mL^−1^) were incubated with BMSCs or RAW264.7 cells at 37 °C for 3 h. Then, the treated cells were washed with PBS and fixed with 4% paraformaldehyde for 15 min. After washing with PBS, DAPI was applied to stain nuclei. A fluorescence microscope (Carl Zeiss Axio Imager 2, Germany) was used to obtain images.

##### Osteogenic Differentiation Assay

BMSCs (three biological replicates per group) were seeded into 48‐well culture plates and cultured in conventional complete medium (*α*‐MEM + 10% FBS + 1% penicillin–streptomycin). On reaching 80% confluence, the medium was replaced with osteogenic medium (Cat. No. MUCMX‐90021; Cyagen Biosciences Inc, Guangzhou, China) supplemented with 6 × 10^8^ vesicles mL^−1^ of different bacterial EVs, 10 µg mL^−1^ non‐EV‐containing fractions, or an equal volume of vehicle (PBS). BMSCs cultured in conventional complete *α*‐MEM medium served as the negative control group. Half of the medium with EVs or vehicle was changed every other day. For analysis of the expression of osteogenesis‐related genes, total RNA from the cells was extracted after 3 days of induction and processed for qRT‐PCR. For analysis of mineralized nodule formation, cells were induced for 10 days and then subjected to ARS staining with 2% ARS solution (pH = 4.2; Solarbio). After washing with PBS, the stained cells were photographed and then observed at 100× magnification under an inverted microscope (Leica). ARS‐positive area in each well was analyzed using Image‐Pro Plus 6 software and shown as a percentage of ARS‐positive area over total well area.

##### Osteoclastic Differentiation Assay

RAW264.7 cells (three biological replicates per group) were plated into 48‐well culture plates at a density of 5 × 10^3^ cells per well and incubated in conventional complete medium (high‐glucose DMEM + 10% FBS + 1% penicillin–streptomycin). 24 h later, the medium was replaced with fresh complete DMEM medium containing RANKL (100 ng mL^−1^; Peprotech, Rocky Hill, USA) supplemented with 6 × 10^8^ vesicles mL^−1^ of different bacterial EVs, 10 µg mL^−1^ non‐EV‐containing fractions or an equivalent volume of vehicle (PBS). RAW264.7 cells cultured in conventional complete DMEM medium were severed as the negative control group. Half of the medium with EVs or vehicle was changed every other day. After 3 days of induction, total RNA from the cells was harvested and subjected to qRT‐PCR for analyzing the expression of osteoclastogenesis‐related genes. After 8 days of induction, the cells were rinsed with PBS and fixed with 4% paraformaldehyde for 10 min. After PBS rinsing, TRAP activities were tested by a TRAP staining kit (Cat. No. 387A‐1KT; Sigma‐Aldrich). TRAP‐positive cells with more than three nuclei were regarded as osteoclasts. The number of osteoclasts in each well was counted under an inverted microscope (Leica). Total osteoclast cell area was analyzed using Image‐Pro Plus 6 software and shown as a percentage of total osteoclast cell area over total well area.

##### 16s rRNA Gene Sequencing

Approximately 0.5 g fecal sample was subject for DNA extraction using the QIAamp Fast DNA Stool Mini Kit (Cat. No. 51604; Qiagen, Hilden, Germany). The DNA samples were sent to GeneSky Biological Technology (Shanghai, China) for analysis of GM composition and quantification of the relative abundance of each member by high‐throughput 16s rRNA gene sequencing. The variable V4 region of 16S rDNA was chosen for amplification and sequenced by Illumina Miseq (Illumina). The quality filtering criteria were a minimum 200 bp in length, minimum quality score of 30, no mismatches in the primers sequence, and no more than six ambiguous bases. USEARCH was used to perform filtering of duplicate sequences and chimera removal. The remaining sequences were clustered into operational taxonomic units (OTUs) with a 97% similarity threshold using the UCLUST algorithm and then classified against the Greengene database. The relative abundance of the identified microbiota at the phylum, class, order, family, genus, and species levels was shown in a table. The relative abundance of the microbiota at the phylum and genus levels was plotted as bar graphs.

##### qRT‐PCR Analysis

For analysis of *Akk* level by qRT‐PCR, fecal samples were freshly collected and weighed. Fecal genomic DNA was extracted from fecal samples using the QIAamp Fast DNA Stool Mini Kit (Qiagen) and subjected to qRT‐PCR amplification with the specific primers designed from the variable regions of the 16S rRNA gene sequence of *Akk* (forward, 5′‐CCTTGCGGTTGGCTTCAGAT‐3′, and reverse, 5′‐CAGCACGTGAAG GTGGGGAC‐3′) as described previously.^[^ ^48^ ^]^ The amplification reactions were carried out in 10 µL reaction volumes containing primers and FastStart Universal SYBR Premix ExTaq (Takara Biotechnology, Japan), and were performed in FTC‐3000 real‐time PCR system (Funglyn Biotech Inc., Toronto, Canada). The cycle threshold of each sample was compared with a standard curve (performed in duplicate) made by diluting genomic DNA from *Akk*. The data were expressed as log_10_ of *Akk* number per gram of fecal content.

For analysis of the expression of osteogenesis‐ or osteoclastogenesis‐related genes, total cellular RNA of BMSCs and RAW264.7 cells in different treatment groups was isolated using TRIzol Reagent (Invitrogen) and reverse‐transcribed into cDNA with All‐in‐One cDNA Synthesis SuperMix (Biotool, Houston, USA), followed by qRT‐PCR using FTC‐3000 real‐time PCR system. *Gapdh* served as an internal control. Primers for qRT‐PCR are shown in Table S5 in the Supporting Information.

##### Statistical Analysis

All data were presented as mean ± SD. Unpaired, two tailed Student's *t*‐test was used to analyze the differences between two groups. Statistical analysis of multiple‐group comparisons was performed by one‐way analysis of variance (ANOVA), followed by the Bonferroni post hoc test to assess the significance of differences between two groups. For all experiments, *P* < 0.05 was considered to be significant and represented by “^#/*/▼/▲^”; *P* < 0.01 was represented by “^##/**/▼▼/▲▲^”; *P* < 0.001 was represented by “^###/***/▼▼▼/▲▲▲^.”. GraphPad Prism software (Version 6.01) was used for above statistical analysis.

## Conflict of Interest

The authors declare no conflict of interest.

## Author Contributions

J.‐H.L. and C.‐Y.C. contributed equally to this work. J.‐H.L., Z.‐W.L., S.‐S.R., C.‐Y.C., L.J., T.‐F.W., T.Y., Y.‐J.T., H.Y., Y.‐Y.W., K.X., J.C., C.‐G.H., and J.L. performed the experiments. H.X., C.‐Y.C., J.‐H.L., and Z.‐Z.L. designed the experiments. J.‐H.L., Z.‐Z.L., and C.‐Y.C. analyzed the data. J.‐H.L., C.‐Y.C., and Z.‐Z.L. prepared all the figures. F.Y., F.‐Y.H., J.G., Z.‐X.W., M.‐J.L., X.‐K.H., Y.‐W.L., W.D., Y.H., Y.Z., J.H., H.‐M.L., B.W., H.‐M.L., T.‐H.C., Y.‐X.Q., Y.‐Y.L., S.‐K.F., Y.C., L.‐Y.Q., R.X., and S.‐Y.T. provided technical support. H.X., C.‐Y.C., and J.‐H.L. wrote the manuscript.

## Supporting information

Supporting Information

Supplemental Table 2

Supplemental Table 3
