# Supplementary material for: Extracellular Vesicles from Child Gut Microbiota Enter into Bone to Preserve Bone Mass and Strength
Source: Adv Sci (Weinh). 2021 Feb 17;8(9):2004831. doi: 10.1002/advs.202004831 (PMC8097336; doi:10.1002/advs.202004831)
Supplement: Supplementary file 1 — Supporting Information [file ADVS-8-2004831-s002.pdf]

© 2021 Wiley-VCH GmbH

ADVANCED SCIENCE
Open Access

Supporting Information

for *Adv. Sci.,* DOI: 10.1002/advs.202004831

Extracellular vesicles from child gut microbiota enter into bone to preserve bone mass and strength

*Jiang-Hua Liu# Chun-Yuan Chen#, Zheng-Zhao Liu, Zhong-Wei Luo, Shan-Shan Rao, Ling Jin, Teng-Fei Wan, Tao Yue, Yi-Juan Tan, Hao Yin, Fei Yang, Fei-Yu Huang, Jian Guo, Yi-Yi Wang, Kun Xia, Jia Cao, Zhen-Xing Wang, Chun-Gu Hong, Ming-Jie Luo, Xiong-Ke Hu, Yi-Wei Liu, Wei Du, Juan Luo, Yin Hu, Yan Zhang, Jie Huang, Hong-Ming Li, Ben Wu, Hao-Ming Liu, Tuan-Hui Chen, Yu-Xuan Qian, You-You Li, Shi-Kai Feng, Yang Chen, Lu-Yue Qi, Ran Xu, Si-Yuan Tang, Hui Xie***

Supporting Information

Extracellular vesicles from child gut microbiota enter into bone to preserve bone mass and strength

Jiang-Hua Liu[#] Chun-Yuan Chen[#], Zheng-Zhao Liu, Zhong-Wei Luo, Shan-Shan Rao, Ling Jin, Teng-Fei Wan, Tao Yue, Yi-Juan Tan, Hao Yin, Fei Yang, Fei-Yu Huang, Jian Guo, Yi-Yi Wang, Kun Xia, Jia Cao, Zhen-Xing Wang, Chun-Gu Hong, Ming-Jie Luo, Xiong-Ke Hu, Yi-Wei Liu, Wei Du, Juan Luo, Yin Hu, Yan Zhang, Jie Huang, Hong-Ming Li, Ben Wu, Hao-Ming Liu, Tuan-Hui Chen, Yu-Xuan Qian, You-You Li, Shi-Kai Feng, Yang Chen, Lu-Yue Qi, Ran Xu, Si-Yuan Tang, Hui Xie[*]

[#] Jiang-Hua Liu and Chun-Yuan Chen contributed equally to this work.

* Correspondence: Hui Xie: huixie@csu.edu.cn; #87 Xiangya Road, Changsha, Hunan, 410008, China

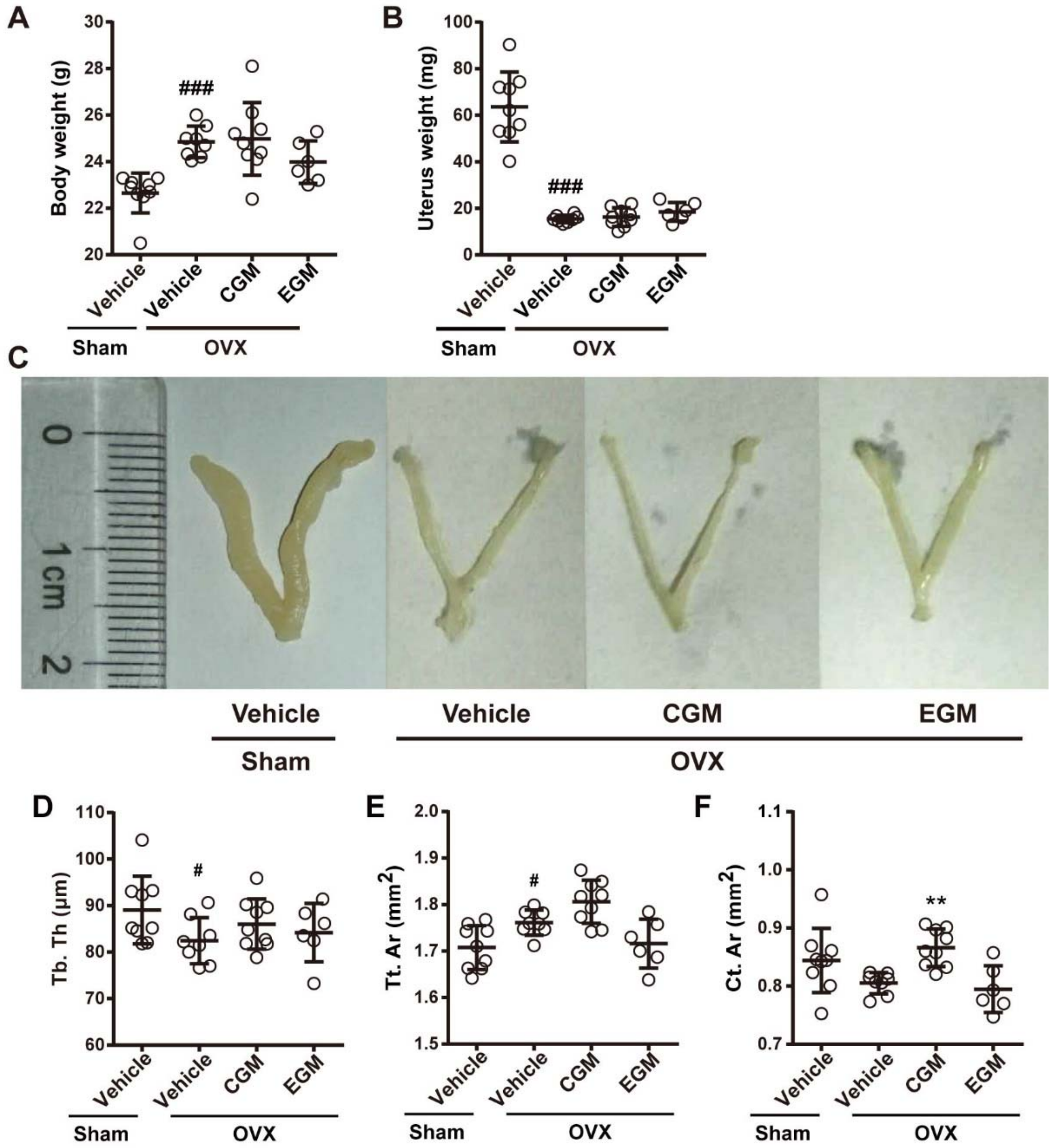

Figure S1. Effects of transplantation with pooled CGM and EGM on body weights, uterus sizes, uterus weights and bone microstructural parameters in OVX mice. A) Body weights of mice in Sham + Vehicle, OVX + Vehicle, OVX + pooled CGM and OVX + pooled EGM groups. *n* = 6-9 per group. B) Quantification of uterus weights. *n* = 6-9 per group. C) Representative images of uteruses from mice receiving different treatments. D-F) Quantitative analysis of trabecular thickness (Tb. Th; D), total cross-sectional area (Tt. Ar; E) and cortical bone area (Ct. Ar; F) in femora from mice receiving different treatments. *n* = 6-9 per group. Data are presented as mean ± SD. $^{\#}P < 0.05$ vs. Sham + Vehicle group, $^{*}P < 0.05$ vs. OVX + Vehicle group. $^{\#}P < 0.05$, $^{**}P < 0.01$, $^{\#\#\#}P < 0.001$.

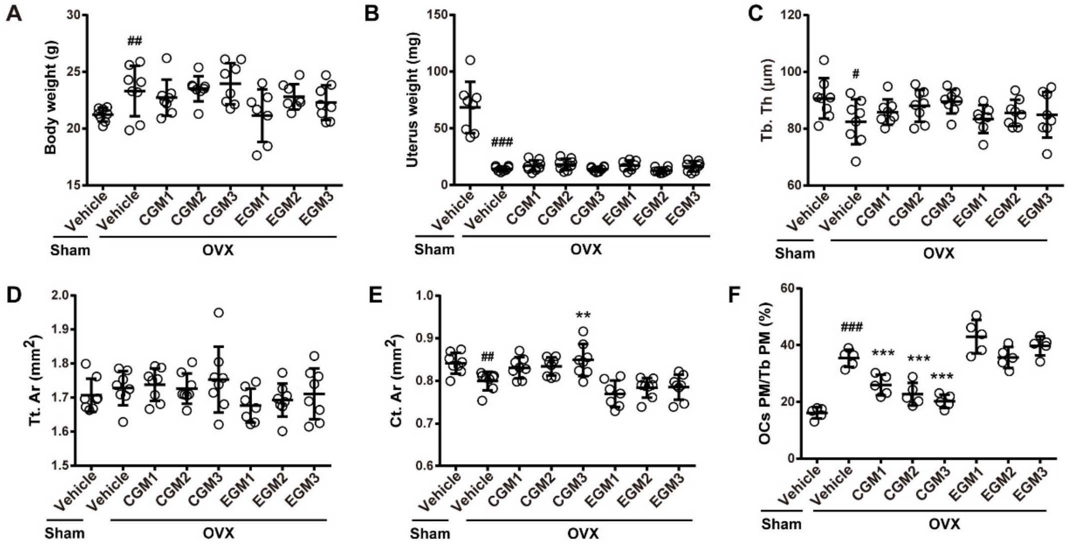

Figure S2. Effects of transplantation with different donors-derived CGM and EGM on body weights, uterus weights, bone microstructural parameters and osteoclast sizes in OVX mice. A, B) Body weights (A) and uterus weights (B) of Sham and OVX mice treated with vehicle or different donors-derived CGM or EGM. n = 7-8 per group. C-E) Quantitative analysis of Tb. Th (C), Tt. Ar (D) and Ct. Ar (E) in femora from Sham and OVX mice receiving different treatments. n = 7-8 per group. F) Quantitative analysis of osteoclast perimeter (OCs PM) per millimeter of trabecular bone perimeter (Tb PM). n = 5 per group. Data are presented as mean ± SD. $^{\#}P < 0.05$ vs. Sham + Vehicle group, $^{*}P <$ 0.05 vs. OVX + Vehicle group. $^{\#}P < 0.05$, $^{\#\#/**}P < 0.01$, $^{\#\#\#/***}P < 0.001$.

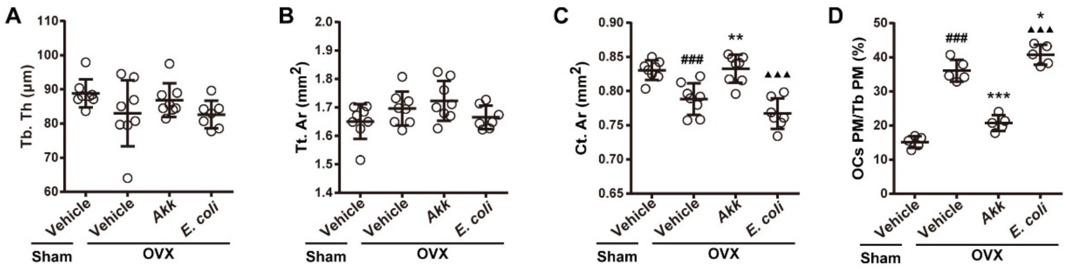

Figure S3. Effects of transplantation with *Akk* on bone microstructural parameters and osteoclast sizes in OVX mice. A-C) Quantitative analysis of Tb. Th (A), Tt. Ar (B) and Ct. Ar (C) in femora from Sham and OVX mice treated with vehicle, *Akk* or *E. coli*. n = 7-8 per group. D) Quantitative analysis of OCs PM/Tb PM. n = 5 per group. Data are presented as mean ± SD. $^{\#}P < 0.05$ vs. Sham + Vehicle group, $^{*}P < 0.05$ vs. OVX + Vehicle group, $^{\blacktriangle}P < 0.05$ vs. OVX + *Akk* group. $^{*}P < 0.05$, $^{**}P < 0.01$, $^{\#\#\#/***/\blacktriangle\blacktriangle\blacktriangle}P <$ 0.001.

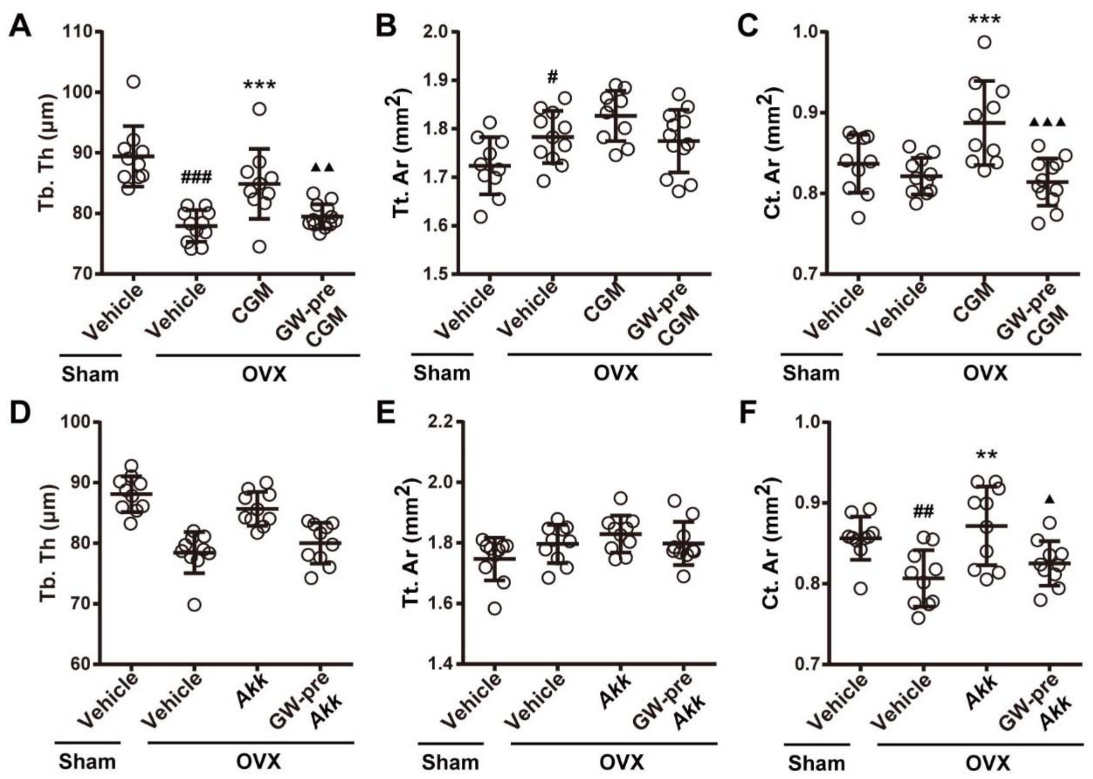

Figure S4. Effects of transplantation with GW4869-pretreated CGM and *Akk* on bone microstructural parameters in OVX mice. A-C) Quantitative analysis of Tb. Th (A), Tt. Ar (B) and Ct. Ar (C) in femora from Sham and OVX mice treated with vehicle, CGM or GW4869-pretreated CGM. n = 10-12 per group. D-F) Quantitative analysis of Tb. Th (D), Tt. Ar (E) and Ct. Ar (F) in femora from Sham and OVX mice treated with vehicle, *Akk* or *Akk*-pretreated CGM. n = 10 per group. Data are presented as mean ± SD. $^{\#}P < 0.05$ vs. Sham + Vehicle group, $^{*}P < 0.05$ vs. OVX + Vehicle group, $^{\blacktriangle}P < 0.05$ vs. OVX + CGM or *Akk* group. $^{\#/\blacktriangle}P < 0.05$, $^{\#\#/**/\blacktriangle\blacktriangle}P < 0.01$, $^{\#\#\#/***/\blacktriangle\blacktriangle\blacktriangle}P < 0.001$.

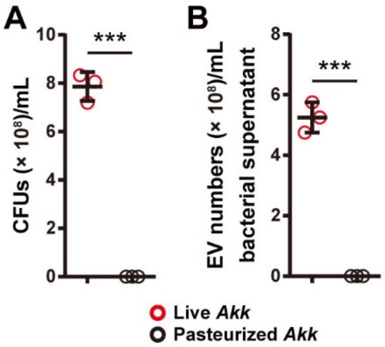

Figure S5. Pasteurization impairs *Akk* viability and EVs secretion. A) Quantification of the number of bacterial colonies formed by the live or pasteurized *Akk*. $n = 3$ per group. B) Particle numbers of EVs from the live or pasteurized *Akk*. $n = 3$ per group. Data are presented as mean ± SD. $^{***}P < 0.001$.

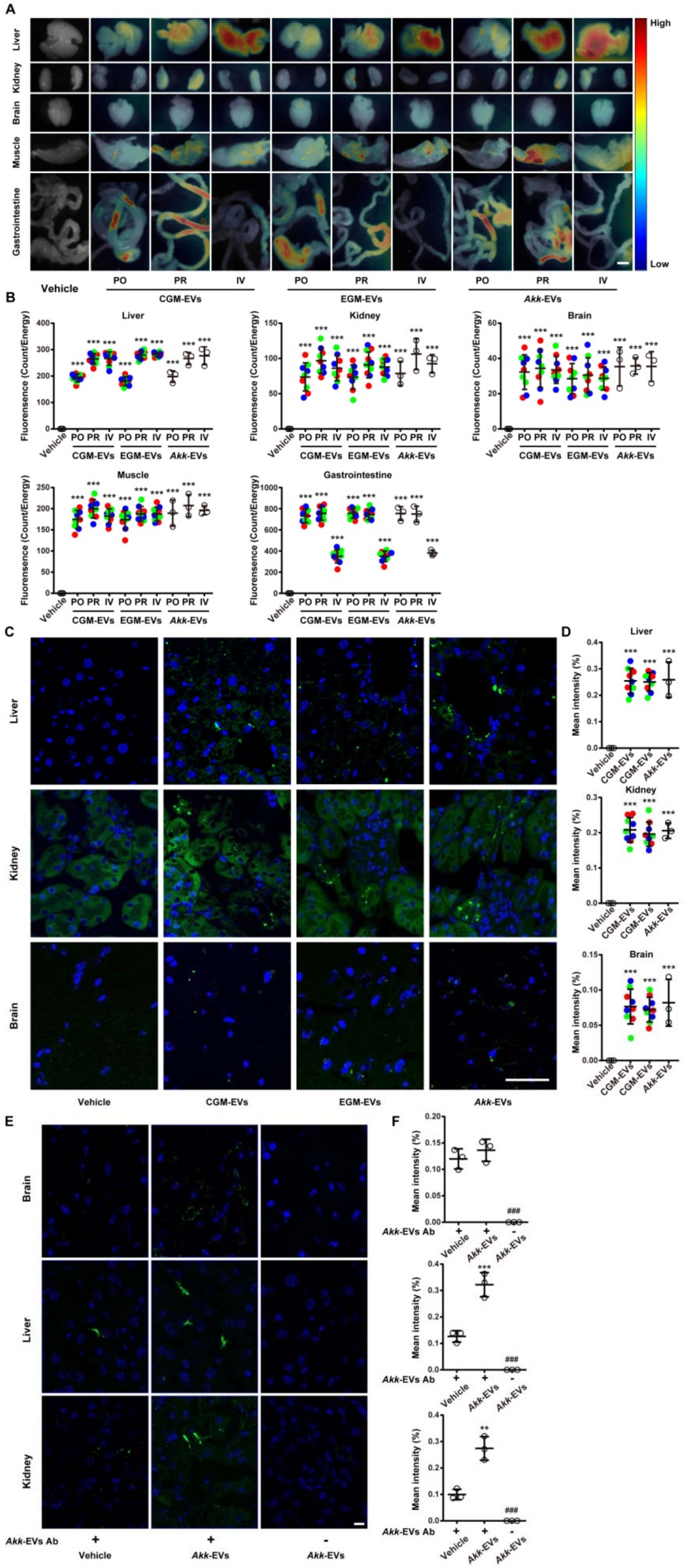

A
Liver
Kidney
Brain
Muscle
Gastrointestine
High
Low
Vehicle
PO
PR
IV
CGM-EVs
EGM-EVs
Akk-EVs
B
Liver
Kidney
Brain
Muscle
Gastrointestine
Fluorensence (Count/Energy)
C
D
Mean intensity (%)
E
F
Akk-EVs Ab

Figure S6. Biodistribution of CGM-EVs and *Akk*-EVs in mice. A, B) *Ex vivo* fluorescent imaging of the muscle, brain, liver, kidney and gastrointestine from mice treated with vehicle or the DIR-labeled EVs for 1 h (A) and quantification of the fluorescent signals (B). PO: *per os*; PR: *per rectal*; IV: intravenous. Scale bar: 6 mm. n = 3 per group. C) Confocal microscopy analysis of the brain, liver and kidney sections from mice treated with the PKH67-labeled EVs for 1 h by oral administration. Scale bar: 50 μm. D) Quantification of the fluorescent signals in (C). n = 3 per group. E, F) Representative images of the *Akk*-EVs antibody (Ab)-stained brain, liver and kidney sections (E) with quantification of the positive signals (F). n = 3 per group. Scale bar: 10 μm. For B and D: Red dots indicate CGM1-EVs or EGM1-EVs, green dots indicate CGM2-EVs or EGM2-EVs, and blue dots indicate CGM3-EVs or EGM3-EVs. Data are presented as mean ± SD. For B, D: $^{*}P < 0.05$ vs. Vehicle group. For F: $^{*}P < 0.05$ vs. Vehicle + *Akk*-EVs Ab group, $^{\#}P < 0.05$ vs. *Akk*-EVs + *Akk*-EVs Ab group. $^{**}P < 0.01$, $^{***/\#\#\#}P < 0.001$.

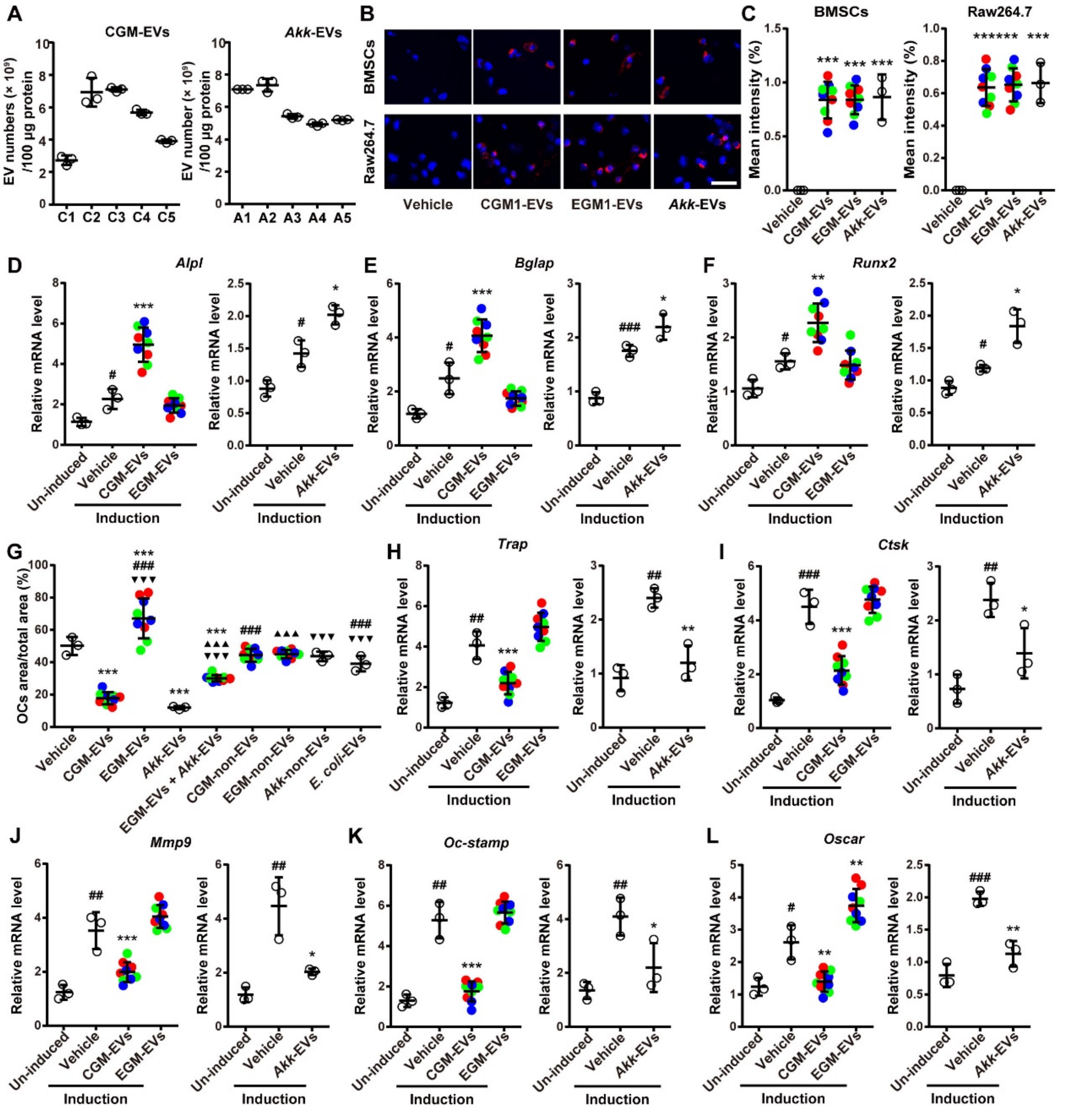

Figure S7. CGM-EVs and *Akk*-EVs directly promote osteogenesis and inhibit osteoclastogenesis *in vitro*. A) Particle numbers of 100 μg CGM-EVs (C1, C2, C3, C4 and C5) and 100 μg *Akk*-EVs (A1, A2, A3, A4 and A5) from different batches. *n* = 3 per group. B, C) Uptake of the PKH26-labeled different EVs by BMSCs and RAW264.7 cells (B), and quantification of the fluorescent signals (C). Scale bar: 100 μm. *n* = 3 per group. D-F) qRT-PCR analysis of the expression levels of osteogenic genes including *Bglap* (D), *Alpl* (E) and *Runx2* (F) in BMSCs receiving different treatments. *n* = 3 per group. G) Quantification of the percentage of total osteoclast cell area over total well area. *n* = 3 per group. H-L) qRT-PCR analysis of the expression levels of osteoclastogenesis-related genes including *Trap* (H), *Ctsk* (I), *Mmp9* (J), *Oc-stamp* (K)

and *Oscar* (L) in RAW264.7 cells receiving different treatments. $n = 3$ per group. For C-L: Red dots indicate CGM1-EVs or EGM1-EVs, green dots indicate CGM2-EVs or EGM2-EVs, and blue dots indicate CGM3-EVs or EGM3-EVs. Data are presented as mean ± SD. For C: $^{*}P < 0.05$ vs. Vehicle group. For G: $^{*}P < 0.05$ vs. Vehicle group, $^{\#}P < 0.05$ vs. CGM-EVs group, $^{▲}P < 0.05$ vs. OVX + EGM-EVs group, $^{▼}P < 0.05$ vs. *Akk*-EVs group. For others: $^{\#}P < 0.05$ vs. Un-induced group, $^{*}P < 0.05$ vs. positive control (Vehicle) group, $^{▲}P < 0.05$ vs. OVX + EGM-EVs group, $^{▼}P < 0.05$ vs. CGM-EVs group. $^{\#/*}P < 0.05$, $^{\#\#/**}P < 0.01$, $^{\#\#\#/***/▲▲▲/▼▼▼}P < 0.001$.

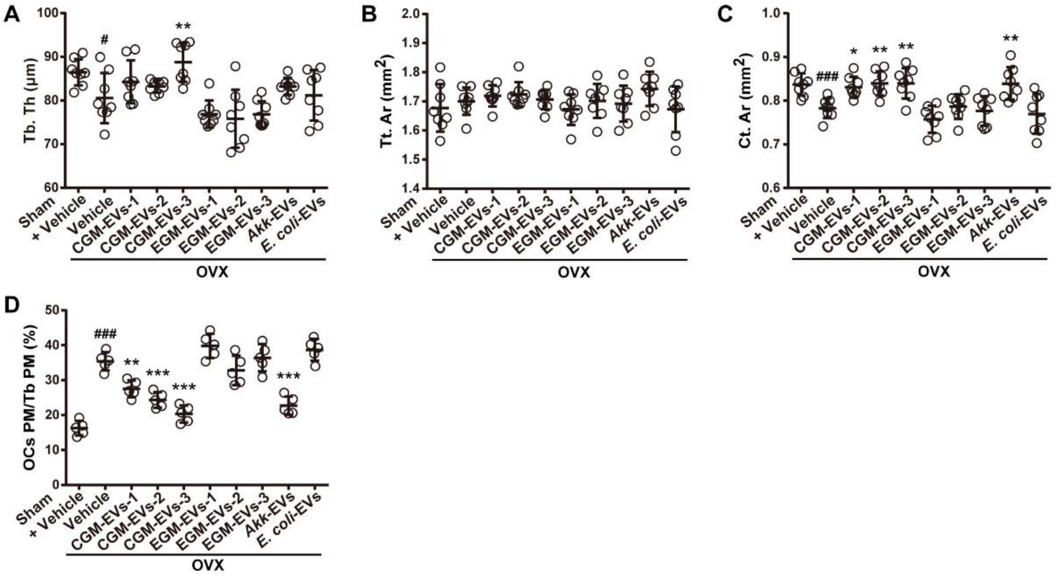

Figure S8. Effects of oral transplantation with different bacterial EVs on bone microstructural parameters and osteoclast sizes in OVX mice. A-C) Quantitative analysis of Tb. Th (A), Tt. Ar (B) and Ct. Ar (C) in femora from Sham and OVX mice treated with vehicle or different EVs by oral route. *n* = 8 per group. D) Quantitative analysis of OCs PM/Tb PM. *n* = 5 per group. Data are presented as mean ± SD. $^{\#}P < 0.05$ vs. Sham + Vehicle group, $^{*}P < 0.05$ vs. OVX + Vehicle group. $^{\#/*}P < 0.05$, $^{**}P < 0.01$, $^{\#\#\#/***}P < 0.001$.

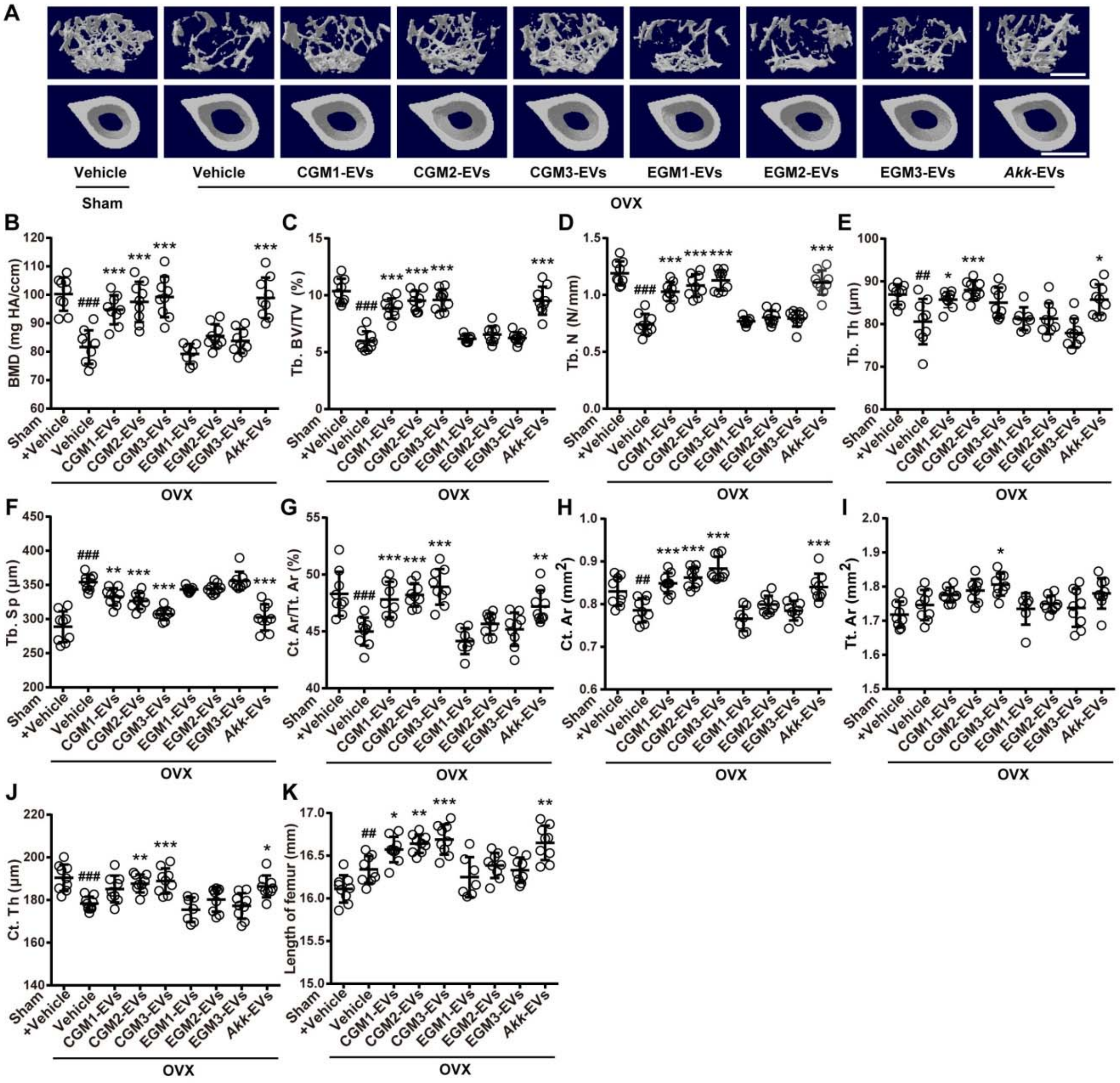

Figure S9. CGM-EVs and *Akk*-EVs increase bone mass and length in OVX mice after intravenous administration. A-J) Representative μCT images of trabecular (up) and cortical (bottom) bone in femora from Sham and OVX mice receiving different treatments by intravenous route (A) and quantification of BMD (B), Tb. BV/TV (C), Tb. N (D), Tb. Th (E), Tb. Sp (F), Ct. Ar/Tt. Ar (G), Ct. Ar (H), Tt. Ar (I) and Ct. Th (J). Scale bar: 500 μm (up) and 1 mm (bottom). *n* = 7-9 per group. K) Lengths of femora. *n* = 7-9 per group. Data are presented as mean ± SD. $^{\#}P < 0.05$ vs. Sham + Vehicle group, $^{*}P < 0.05$ vs. OVX + Vehicle group. $^{*}P < 0.05$, $^{\#\#/**}P < 0.01$, $^{\#\#\#/***}P < 0.001$.

Table S1. Measurements of BMI and blood metabolic indicators in CGM and EGM donors.

Parameters	CGM donors	EGM donors	Reference value	Unit
BMI	16.40 ± 1.39	20.20 ± 2.08	Adult: 18.5-25; Children (3-5-year-old) [a]	kg/m^2
Blood glucose	5.23 ± 0.55	5.99 ± 0.15	3.9-6.1	mmol/L
Total cholesterol	3.76 ± 0.45	5.5 0± 0.40	3.2-5.7	mmol/L
Triglyceride	1.07 ± 0.09	1.23 ± 0.23	0.57-1.7	mmol/L
Total protein	65.73 ± 2.38	69.03 ± 2.10	60-80	g/L
Albumen	44.60 ± 1.23	43.63 ± 2.02	40-55	g/L
Globulin	21.13 ± 1.46	25.40 ± 3.48	20-30	g/L
Albumen/Globulin	2.11 ± 0.13	1.74 ± 0.30	1.2-2.4	

Data are presented as mean ± SD. a: The BMI values (mean ± SD) of 3-5-year-old Chinese boys and girls in the 5th NSPGDC (The National Survey on the Physical Growth and Development of Children in the Nine Cities of China) are displayed as follows (DOI: 10.1002/ajpa.23224):

3-year-old: boy, 15.63 ± 1.32; girl, 15.38 ± 1.29

4-year-old: boy, 15.53 ± 1.45; girl, 15.22 ± 1.37

5-year-old: boy, 15.58 ± 1.68; girl, 15.21 ± 1.57

Table S4. Serum antibody titer against *Akk*-EVs tested by ELISA.

**Dilution**	**OD values (*Akk*-EVs from rabbit-1)**	**OD values (*Akk*-EVs from rabbit-2)**
1:10,000	2.463	2.344
1:50,000	2.262	1.613
1:250,000	1.861	0.613
1:1,250,000	0.814	0.333
1:6,250,000	0.284	0.126
1:31,250,000	0.114	0.043
1:156,250,000	0.078	0.056
Negative control	0.011	0.017
Antibody titer	**1:3.125×10^{7}**	**1:6.25×10^{6}**

Table S5. Primer sequences for qRT-PCR.

Gene	Forward (5'-3')	Reverse (5'-3')
Bglap	CTGACCTCACAGATCCCAAGC	TGGTCTGATAGCTCGTCACAAG
Alpl	CCAACTCTTTTGTGCCAGAGA	GGCTACATTGGTGTTGAGCTTTT
Runx2	GACTGTGGTTACCGTCATGGC	ACTTGGTTTTTCATAACAGCGGA
Trap	TGGTCCAGGAGCTTAACTGC	GTCAGGAGTGGGAGCCATATG
Ctsk	GCGGCATTACCAACAT	CTGGAAGCACCAACGA
Mmp9	ACCCGAAGCGGACATT	GGCATCTCCCTGAACG
Ocstamp	GGGCTACTGGCATTGCTCTTAGT	CCAGAACCTTATATGAGGCGTCA
Oscar	GGTCCTCATCTGCTTG	TATCTGGTGGAGTCTGG
Gapdh	CACCATGGAGAAGGCCGGGG	GACGGACACATTGGGGGTAG
